# Evolutionary divergent clusters of transcribed extinct truncated retroposons drive low mRNA expression and developmental regulation in the protozoan *Leishmania*

**DOI:** 10.1186/s12915-024-02051-4

**Published:** 2024-10-29

**Authors:** Gabriel Reis Ferreira, Jean-Guillaume Emond-Rheault, Lysangela Alves, Philippe Leprohon, Martin A. Smith, Barbara Papadopoulou

**Affiliations:** 1 Research Center in Infectious Diseases and Axis of Infectious and Immune Diseases, Research Center of the Centre Hospitalier Universitaire de Québec-Université Laval, QC Quebec, Canada; 2 Department of Microbiology, Infectious Disease and Immunology, Faculty of Medicine, University Laval, Quebec, QC G1V 4G2 Canada; 3 Rua Prof. Algacyr Munhoz Mader 3775, Curitiba/PR, CIC 81310-020 Brazil; 4 CHU Sainte-Justine Research Centre, Montreal, QC H3T 1C5 Canada; 5 Department of Biochemistry and Molecular Medicine, Faculty of Medicine, University of Montreal, QC Montreal, H3T 1J4 Canada; 6 School of Biotechnology and Molecular Bioscience, Faculty of Science, UNSW Sydney, NSW Sydney, 2052 Australia

**Keywords:** *Leishmania infantum*, SIDER2 retroposons, Evolutionary divergence, Chromosome clustering, SIDER2 transcriptome, Low expression, Developmental gene regulation, Virulence, Functional bias

## Abstract

**Background:**

The *Leishmania* genome harbors formerly active short interspersed degenerated retroposons (SIDERs) representing the largest family of repetitive elements among trypanosomatids. Their substantial expansion in *Leishmania* is a strong predictor of important biological functions. In this study, we combined multilevel bioinformatic predictions with high-throughput genomic and transcriptomic analyses to gain novel insights into the diversified roles retroposons of the SIDER2 subfamily play in *Leishmania* genome evolution and expression.

**Results:**

We show that SIDER2 retroposons form various evolutionary divergent clusters, each harboring homologous SIDER2 sequences usually located nearby in the linear sequence of chromosomes. This intriguing genomic organization underscores the importance of SIDER2 proximity in shaping chromosome dynamics and co-regulation. Accordingly, we show that transcripts belonging to the same SIDER2 cluster can display similar levels of expression. SIDER2 retroposons are mostly transcribed as part of 3'UTRs and account for 13% of the *Leishmania* transcriptome. Genome-wide expression profiling studies underscore SIDER2 association generally with low mRNA expression. The remarkable link of SIDER2 retroposons with downregulation of gene expression supports their co-option as major regulators of mRNA abundance. SIDER2 sequences also add to the diversification of the *Leishmania* gene expression repertoire since ~ 35% of SIDER2-containing transcripts can be differentially regulated throughout the parasite development, with a few encoding key virulence factors. In addition, we provide evidence for a functional bias of SIDER2-containing transcripts with protein kinase and transmembrane transporter activities being most represented.

**Conclusions:**

Altogether, these findings provide important conceptual advances into evolutionary innovations of transcribed extinct retroposons acting as major RNA cis-regulators.

**Supplementary Information:**

The online version contains supplementary material available at 10.1186/s12915-024-02051-4.

## Background

*Leishmania* spp. cause leishmaniases, a large spectrum of complex diseases that infect more than 12 million people in ~ 98 countries with > 350 million people currently at risk [1]. Leishmaniasis is a major public health problem, but its control is hampered by the absence of vaccines and limited, toxic, or ineffective drugs due also to the emergence of drug resistance [2]. *Leishmania* is transmitted to mammals as metacyclic promastigotes by infected phlebotomine sandflies. Once phagocytosed by mammalian macrophages, they differentiate into amastigote forms which replicate in the phagolysosome, an acidic environment that normally digests pathogens. Within macrophages, *Leishmania* encounters drastic environmental changes that shape adaptive responses to stress and trigger stage-specific regulation, hence enabling the parasite to survive and to cause disease.

*Leishmania* is an early-branching unicellular eukaryote that displays unusual features regarding its regulation of gene expression. Unlike many other eukaryotes, *Leishmania* lacks individual control of transcription initiation. Instead, transcription initiated by RNA polymerase II is polycistronic, and individual mRNAs are generated by coupled 5'-trans splicing and 3'-polyadenylation cleavages [3, 4]. Thus, control of gene expression relies almost exclusively on posttranscriptional regulation (PTR) that governs pre-mRNA processing, mRNA decay, and translation rates throughout the parasite development [4–6]. PTR is pivotal to the adaptive responses and survival of *Leishmania* within its insect and mammalian hosts [7, 8]. Cis-acting elements within 3'-untranslated regions (3'UTRs) of *Leishmania* transcripts have been shown to play a central role in PTR. We [5, 9–16] and others [17–20] have identified several classes of 3'UTR elements in *Leishmania* contributing to either mRNA stability or translation. We have also described a new paradigm of PTR in trypanosomatids driven by 3'UTR-located retroposon elements [12].

*Trypanosoma* and *Leishmania* species harbor long-terminal repeat (LTR) and non-LTR retrotransposons (class-I TEs), but no DNA transposons, which impact genome function and evolution. *Trypanosoma brucei* and *T. cruzi* contain long autonomous retroposons of the ingi clade (ingi and L1Tc, respectively) and short non-autonomous retroposons (ribosomal mobile element/RIME and NARTc), as well as degenerated ingi-related retroposons lacking coding capacity (DIREs) [21–23]. The ingi/RIME and LITc/NARTc retroposon pairs are considered analogous to the human LINE1/Alu and plant LINE/SI pairs [24]. In striking contrast, *Leishmania* species only contain remnants of extinct ingi/L1Tc-like retroposons (DIREs) [25] and noncoding truncated elements (SIDERs). Short interspersed degenerated retroposons (SIDERs) were initially identified in the *L. major* genome assembling two distinct large subfamilies, SIDER1 and SIDER2, which display all the hallmarks of trypanosomatid retroposons [12, 26, 27]. SIDERs represent the most abundant retroposon family described in trypanosomatids and have preferentially expanded in *Leishmania* species; they are ~ 70 times more abundant in *L. major* than in *T. brucei* [12, 26]. We have shown previously that members of the SIDER1 subfamily positively regulate translation rates [13] while members of the SIDER2 subfamily promote co-translational mRNA decay initiated by endonucleolytic cleavage through a deadenylation-independent mechanism [28–30]. SIDERs represent the first example of domestication and expansion of retroposon elements in eukaryotes that have evolved to fulfill critical regulatory cellular functions. However, our current understanding of how SIDERs globally regulate gene expression throughout the parasite development remains fragmentary.

In this study, we combined multilevel bioinformatic predictions with high-throughput genomic and transcriptomic analyses to gain novel insights into the diversified roles the large family of SIDER2 retroposons play in *Leishmania* genome evolution and expression. Our data illustrate the high evolutionary divergence of SIDER2 sequences and their intriguing chromosomal organization and proximity sequence relationship, which may favor functional clustering, chromosome dynamics, and co-regulation. Genome-wide expression profiling studies uncover a major role of SIDER2 elements in monitoring mRNA abundance in a constitutive or stage-specific manner and underscore a broad association of SIDER2 sequences with low mRNA expression. In addition, we provide evidence for a functional bias of SIDER2-bearing transcripts and the presence of SIDER2 sequences in the 3'UTR of several transcripts encoding key *Leishmania* virulence factors. Altogether, these findings highlight the outstanding features of widespread SIDER2 retroposons in shaping *Leishmania* genome evolution and controlling gene expression.

## Results

### Re-alignment and automated annotation of SIDER2 retroposons across the Leishmania infantum genome underline their widespread genomic distribution in non-coding regions

**Fig. 1:**
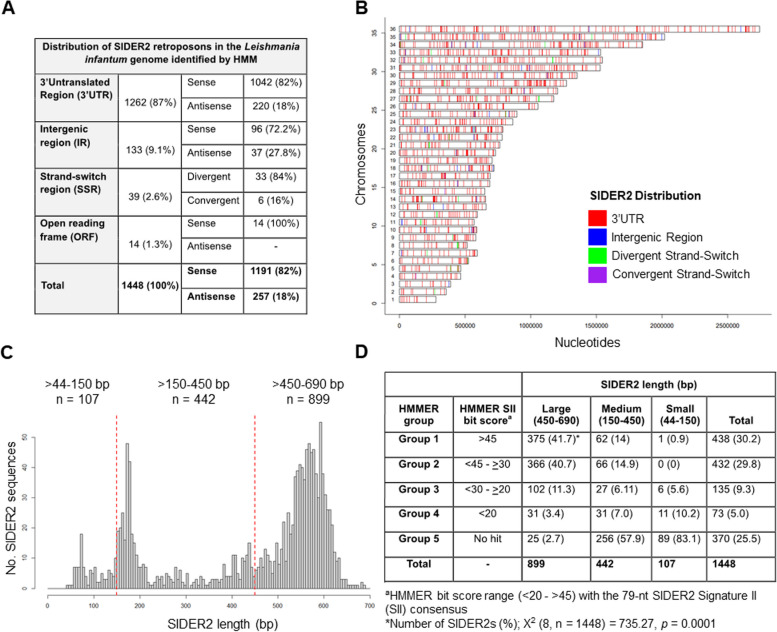
Genomic distribution and evolutionary divergence of SIDER2 retroposons across the *Leishmania infantum* genome. **A** SIDER2 elements were identified by HMM according to their genomic position and orientation relative to the direction of transcription. **B** Schematic diagram showing all the 36 *L. infantum* chromosomes with SIDER2 respective locations highlighting the widespread distribution of SIDER2 retroposons in a synthetic manner. The 3'UTR localization of SIDER2 elements was predicted using the PRED-A-TERM algorithm. **C** Length distribution of SIDER2 elements in *L. infantum*. The graph represents the high length diversity of SIDER2 retroposons (from 44 to 690 bp) divided into three major groups: large-size (> 450–690 bp), medium-size (≥ 150–450 bp), and small-size (> 44–150 bp). **D** Contingency table comprising the HMMER and SIDER2 length groups. Each cell represents the frequency of SIDER2 elements and its respective percentage in parenthesis. The HMMER group is based on the bit score (see “Methods”) with the 79-nt Signature II consensus (group 1 ≥ 45; group 2 < 45 and ≥ 30; group 3 < 30 and ≥ 20; group 4 < 20 and group 5 (no hit)

The SIDER1 and SIDER2 retroposon subfamilies were initially identified in *L. major* [12] and partially characterized in *L. infantum* and *L. braziliensis* where emphasis was given on the SIDER1 subfamily [26]. To carry out an in-depth genomic characterization of the SIDER2 subfamily in *L. infantum*, we used the core SIDER2 consensus sequence encoded in a profile hidden Markov model (HMM) to re-scan for SIDER2 elements across a recent assembly of the *L. infantum* genome (TriTrypDB-62_LinfantumJPCM5) and to perform multiple sequence alignments. The updated and highly sensitive annotation generated a detailed distributional map, uncovering 1448 SIDER2 elements in the *L. infantum* genome in comparison to 1073 initially described in *L. major* [12, 31]. Using a previously developed algorithm (PRED-A-TERM for PREDicting poly(A) sites and TERMinal splice junctions) to effectively predict trans-splicing and poly(A) + sites of *Leishmania* transcripts [32], we mapped 1262 SIDER2 sequences within 3'UTRs (87%) of which 82% were in the sense orientation of transcription and 18% in the antisense (Fig. 1A). One hundred thirty-three SIDER2 elements (9.1%) were found in intergenic regions, 39 (2.6%) in strand-switch regions (SSRs) with most of them located in divergent SSRs (two opposing TSRs: transcription start regions), and 14 SIDER2s were partially overlapping with ORFs coding for hypothetical proteins (Fig. 1A). All 1448 SIDER2 elements were regularly dispersed along the 36 *Leishmania* chromosomes (Fig. 1B). The complete list containing the HMM analysis and the respective chromosomal positions of SIDER2 elements in the *L. infantum* genome is described in Additional file 1: Table S1.

### SIDER2 sequences display a high length variation with the majority maintaining retroposon hallmark sequences at their 5'-end

The 1448 SIDER2 sequences identified in the *L. infantum* genome vary in length from 44 to 690 bp, with a mean of 438 bp and a median of 529 bp (Fig. 1C). Two-thirds of SIDER2 sequences (899 SIDER2s) belong to the large size group ranging from 450 to 690 bp. The medium-size group ranging from 151 to 450 bp regroups 442 SIDER2 elements and the small-length group includes 107 SIDER2 sequences ranging from 44 to 150 bp (Fig. 1C). The large-size SIDER2 group harbors the hallmark sequences of trypanosomatid ingi/RIME and L1Tc/NARTc retroposons, including the 79-nt signature II (SII) at the 5'-end and an adenosine (A)-rich tail at the 3'-end (Additional file 2: Fig. S1). SIDER2 sequences of the medium and small size groups seem to lack the 3'-A-rich tail (with a few exceptions). To determine if the shorter SIDER2s (medium and small size groups) have preserved the 79-nt hallmark sequence, we scanned for the SII consensus using the HMMER software, subsequently establishing similarity cutoffs. HMMER groups were classified based on bit scores in groups 1 (≥ 45), 2 (< 45 to ≥ 30), 3 (< 30 to ≥ 20), 4 (< 20), and 5 (no hits) (Fig. 1D, see also “Methods” for details). Despite their high length variation, the majority of SIDER2 elements (> 70%) share different degrees of homology with the SII consensus. The highest homology (groups 1–3) was detected in the large size SIDER2 group (Fig. 1D). Multiple sequence alignments of randomly selected SIDER2 sequences to the SII consensus using *hmmalign* indicated also a sequence conservation of SII among HMMER groups 1–3 (Additional file 2: Fig. S1B, C). There are however 370 SIDER2 sequences (group 5) belonging mostly to the medium- and small-size groups which seem to have lost SII sequence or harbor degenerated SII that no hits could be found (Fig. 1D). These data highlight the vast length variation between SIDER2 elements in *L. infantum* and indicate that truncated shorter SIDER2s seem to be derived from deletions of the 3'-end of larger elements so to preserve the hallmark 79-nt sequence at their 5'-end.

### SIDER2 retroposons form various evolutionary divergent clusters with each cluster harboring homologous SIDER2 sequences nearby on the same chromosome

**Fig. 2:**
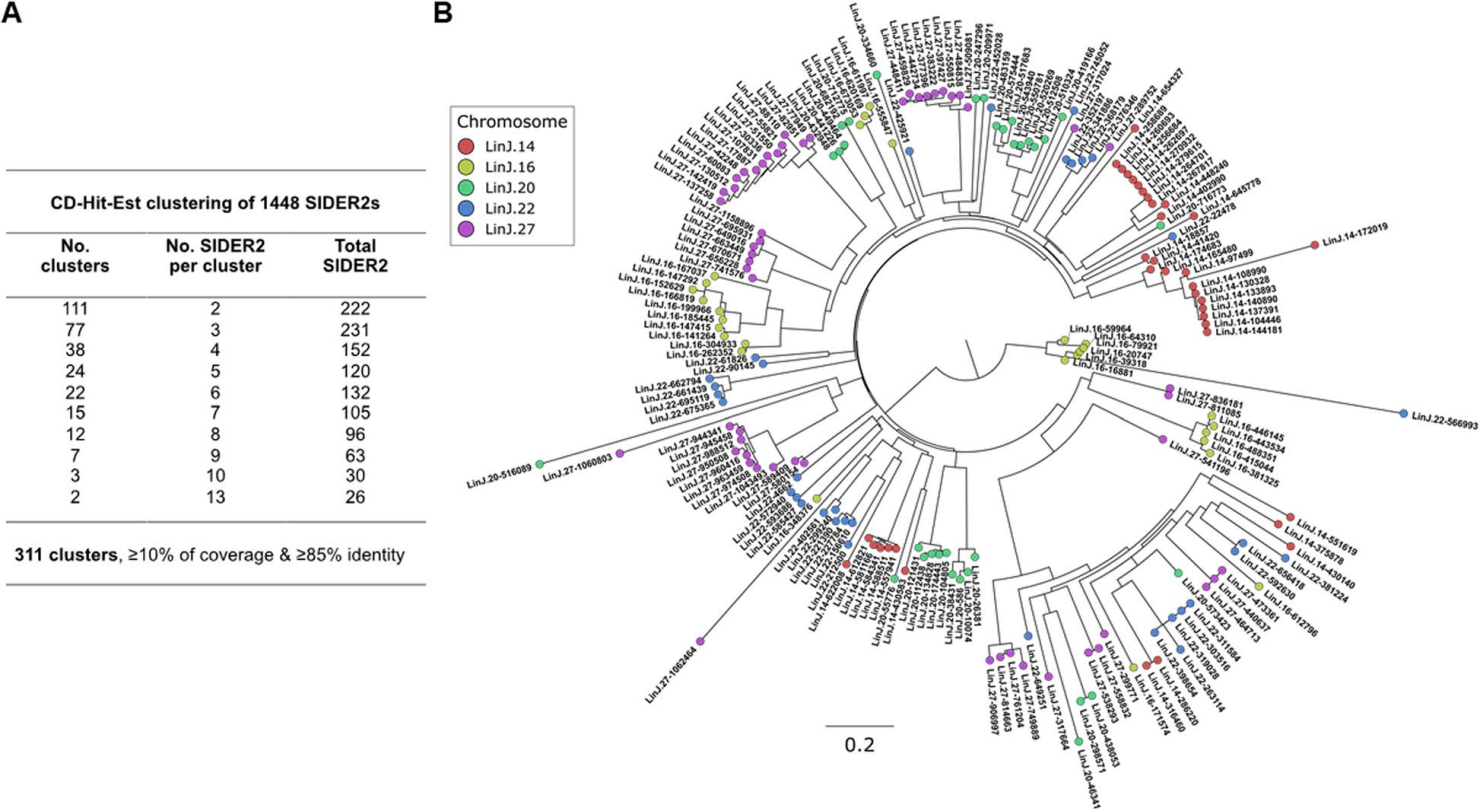
Chromosomal proximity relationship of SIDER2 elements within various SIDER2 clusters. **A** CD-Hit-Est clustering of 1448 SIDER2 sequences based on nucleotide similarity. The columns display the count of clusters, the number of SIDER2 sequences per cluster and the cumulative total of SIDER2 sequences within each category. This clustering analysis employed an adjusted threshold for similarity wherein sequences with ≥ 10% coverage and ≥ 85% sequence identity was considered comparable. **B** Unrooted maximum likelihood tree using IQ-TREE (V.2.1.2) model GTR + I + G1 of 189 SIDER2 sequences from five *L. infantum* chromosomes (LinJ.14, LinJ.16, LinJ.20, LinJ.22, and LinJ.27) aligned by MAFFT (V.7.471) (see “Methods”). Tips were color-coded based on their chromosomal location. Each SIDER2 element is labeled with its corresponding LinJ ID

To further our investigation on SIDER2 sequence composition and chromosomal organization, we used the CD-HIT-Est clustering algorithm to align the 1448 SIDER2 sequences identified from the refined HMM profiles. Clustering analysis employed an adjusted threshold for similarity wherein sequences with ≥ 10% coverage and ≥ 85% sequence identity was considered for comparison. The choice of ≥ 10% coverage CD-HIT-Est threshold (-aL) was motivated by the high size diversity of SIDER2s, as the aim was to cluster also the smaller SIDER2 sequences. Even with ≥ 10% sequence coverage, the vast majority of SIDER2 sequences with a recent common ancestor clustered together using CD-HIT-Est (see Additional file 2: Fig. S2). This analysis identified a total of 311 clusters harboring from 2 to 13 SIDER2 sequences each and only 271 SIDER2 sequences did not cluster based on the above criteria (Fig. 2A and Additional file 1: Table S1).

As part of an independent validation, we performed multiple sequence alignments of the 1448 SIDER2 sequences with MAFFT and evaluated their evolutionary distance using the IQ-TREE model. Phylogenetic analysis demonstrated a high evolutionary divergence between SIDER2 sequences in the *Leishmania* genome and revealed a remarkable SIDER2 chromosomal proximity relationship (Fig. 2B and Additional file 2: Fig. S2A). Most of the SIDER2 clusters (275 clusters; 88.4%) contain SIDER2 sequences that are located on the same chromosome. There are only 36 clusters where SIDER2 sequences are located on different chromosomes (the vast majority on two different chromosomes) (Additional file 1: Table S1). As illustrated in Fig. 2B from the analysis of five randomly selected *L. infantum* chromosomes (Chr14, 16, 20, 22, and 27), each SIDER2 cluster harbors generally nearby SIDER2 sequences on the same chromosome (Fig. 2B and Additional file 2: Fig. S2A). Additionally, a heatmap ordering 189 SIDER2 sequences based on their position on LinJ chromosomes 14, 16, 20, 22, and 27 and aligned with BLASTn (≥ 80% identity) demonstrated that SIDER2 sequences located nearby in the linear sequence of the chromosome share generally higher homology than SIDER2s located further away (Additional file 1: Table S1 and Additional file 2: Fig. S3). We have also investigated if these clusters of phylogenetically close SIDER2 sequences were located within the same posttranscriptional unit (PTU) or beyond PTUs. In Additional file 2: Fig. S3, we can see that from the five chromosomes analyzed, SIDER2 sequences belonging to the same cluster were often found within the same PTU, but there were also examples where a SIDER2 cluster was shared between two different PTUs.

Since nearby SIDER2 elements on the same chromosome are more related, we investigated whether locus-specific SIDER2 clustering was the result of gene duplication events. Thus, we screened the SIDER2-containing transcripts for paralogs with CD-Hit-Est using strict paralog criteria set to ≥ 85% similarity over ≥ 90% of the amino acid sequence length. We detected 47 paralogs comprised in 19 clusters, which represent only 4.2% of *L. infantum* SIDER2-containing transcripts (Additional file 1: Table S2). Interestingly, 17 of the 19 clusters harbor successive paralogous genes on the same chromosome, of which nine were directly contiguous to each other and eight were distanced between 1 and 21 genes (Additional file 1: Table S2). Thus, nearby chromosome clustering of SIDER2 sequences is not the result of gene duplication events, but instead, SIDER2 sequences are mostly found in the 3'UTRs of non-paralogous genes. Combined, these data demonstrate the high evolutionary divergence between SIDER2 sequences in the *Leishmania* genome and highlight the sequence relationship between nearby SIDER2 elements on the same chromosome.

### SIDER2-containing transcripts within the same cluster can display similar levels of expression

**Fig. 3:**
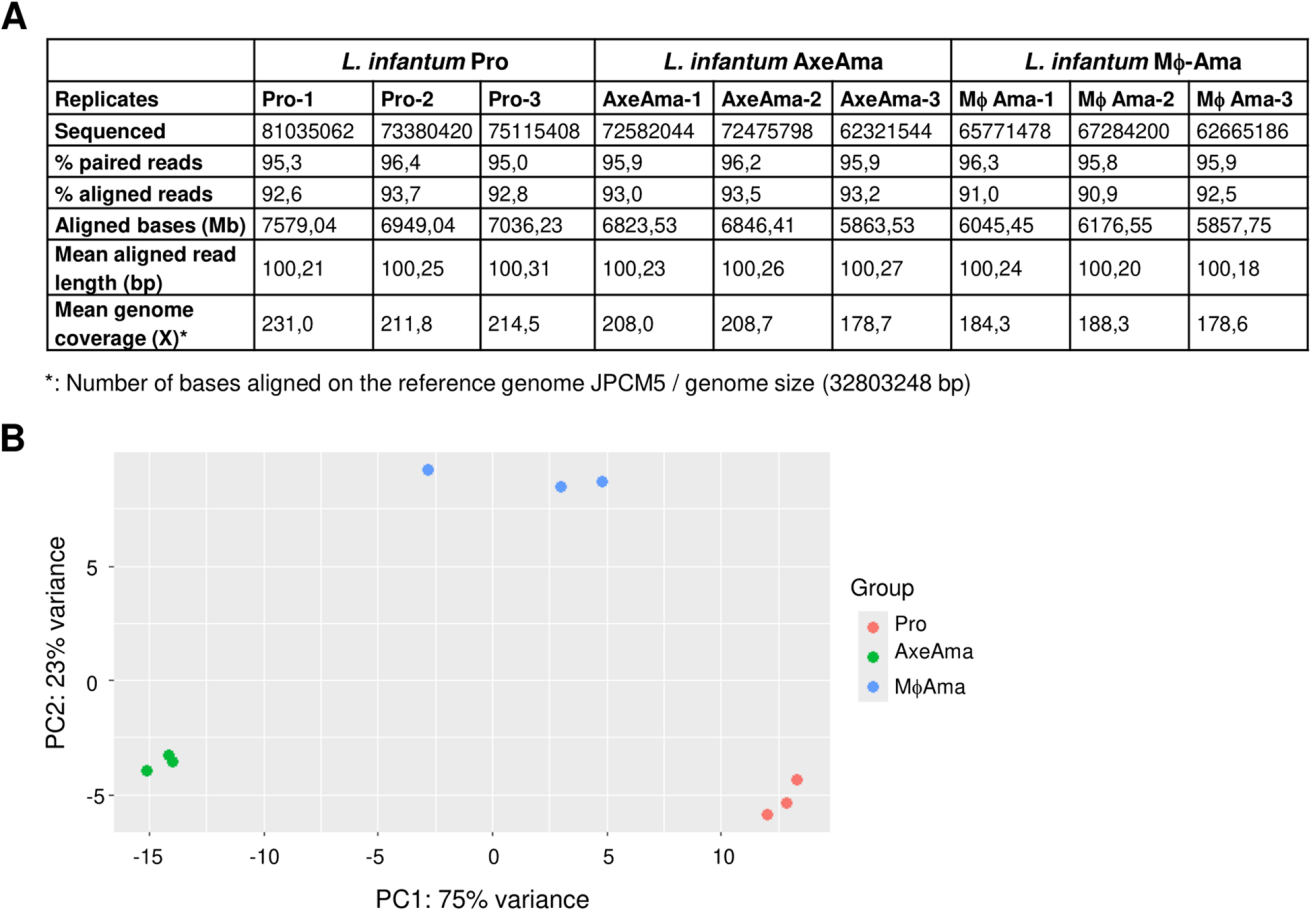
Overall assessment of the Illumina RNA-seq data analysis for *L. infantum* promastigotes, axenically cultured amastigotes, and macrophage-derived amastigotes. Three biological samples were analyzed for each parasite stage. Principal component analysis (PCA) was performed using fragments per kilobase per millions (FPKM) values of all genes. **A** Table resuming Illumina RNA-Seq metrics for each developmental stage and replicates sequenced and aligned. **B** PCA analysis comparing promastigotes (Pro) (in red), axenic amastigotes (AxeAma) (in green), and macrophage-derived amastigotes (MϕAma) (in blue)

**Fig. 4:**
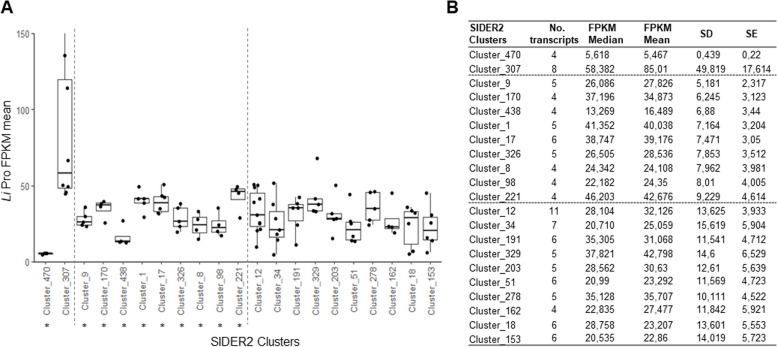
SIDER2 clusters displaying similar levels of expression. Expression levels (based on FPKM mean values) from 74 clusters with ≥ 4 transcripts harboring a single SIDER2 element in their 3'UTR were analyzed here. Transcripts were considered to have a similar expression when the standard deviation (SD) between FPKM mean values was ≤ 10. **A** SIDER2 clusters with most of their SIDER2-containing transcripts exhibiting similar levels of expression in *L. infantum* promastigotes (from 9 to 221). Two clusters with paralogous genes sharing the same expression were included as controls (clusters 470 and 307). In addition, 10 SIDER2 clusters harboring subgroups of two or three transcripts displaying similar expression levels are shown here (clusters 12 to 153). **B** Table summarizing the statistical information for SIDER2 clusters exhibiting similar levels of expression

Sequence-based clustering analysis with CD-HIT-Est revealed that SIDER2 retroposons form a huge variety of evolutionary divergent groups. However, SIDER2s within each cluster share high sequence homology, implying that they can in principle contribute to the same mode of regulation. To be considered co-regulated, transcripts belonging to the same SIDER2 cluster should exhibit similar levels of expression under well-defined growth conditions. To examine this possibility, we analyzed high throughput Illumina poly(A) + RNA sequencing data starting from *L. infantum* promastigotes (see Fig. 3A for details on RNA-seq data) and evaluated expression levels based on FPKM mean values for 74 clusters, each containing ≥ 4 SIDER2 transcripts, from which paralogous genes were subtracted to remove the redundancy attributable to gene duplication. We considered SIDER2-bearing transcripts within each cluster to have a similar expression when the standard deviation (SD) from their FPKM values was < 10 (Additional file 1: Table S2). SD provides a consistent method for performing absolute comparisons. Moreover, it favors assigning similar expression for lowly expressed transcripts as is the case for the majority of SIDER2 transcripts. Using these criteria, we identified nine clusters (clusters 1, 8, 9, 17, 98, 170, 221, 326, 438) with most (> 80%) of their SIDER2-containing transcripts exhibiting similar levels of expression in *L. infantum* grown as promastigotes in culture (Fig. 4A–B**)**. There are also additional examples of SIDER2 clusters (> 25; 10 shown in Fig. 4) harboring subgroups of two or three transcripts that display similar expression levels under promastigote growth conditions (see Fig. 4A–B and Additional file 1: Table S2). Most of these clusters contain transcripts encoding hypothetical proteins and when the function is predicted, it is difficult to conclude whether individual genes per cluster are involved in similar or complementary pathways.

### Genome-wide expression profiling of SIDER2-containing transcripts in both developmental stages of the parasite underscores an association of SIDER2s with low mRNA expression

To establish a solid reference foundation for SIDER2-containing transcripts (“SIDER2ome”), we combined the PRED-A-TERM computational tool (32) with Illumina deep RNA-sequencing data. Thus, we identified 1127 mRNAs harboring SIDER2 elements in their 3'UTR (Additional file 1: Table S3), which accounts for ~ 13% of the *L. infantum* transcriptome. Most of these transcripts (1008; 90%) harbor a single SIDER2 in their 3'UTR, whereas 119 transcripts have two or more SIDER2 sequences (Additional file 1: Table S4). Interestingly, we found that 3'UTRs harboring SIDER2 elements were significantly longer in average (~ 1.8–3 kb) compared to those lacking SIDER2 (~ 0.5–0.8 kb) (*p* < 0.01) (Additional file 2: Fig. S4). Their length was indeed greater than the average size of SIDER2 retroposons (529 bp), with the low expressed SIDER2-bearing transcripts having the longer 3'UTRs (~ 3 kb).

Our previous studies in *L. major* promastigotes using a customized DNA oligonucleotide-based microarray including only 38 SIDER2 transcripts showed that they were less-expressed compared to transcripts lacking SIDER2 sequences [12]. Furthermore, reporter essays established a critical role for SIDER2 sequences in mRNA degradation [28, 33]. To assess whether SIDER2 elements were generally associated with downregulation of gene expression, we analyzed high throughput Illumina sequencing data of poly(A) + RNA isolated from *L. infantum* promastigotes, parasites cultured under conditions triggering amastigote differentiation (increased temperature and low pH; axenic amastigotes) and intracellular amastigotes isolated from THP1-infected macrophages. The average number of total cDNA reads aligned to the reference genome (*L. infantum* JPCM5 V64) among all replicates was ~ 70 million, representing a 200 × coverage (Fig. 3A). StringTie successfully identified aligned reads for 99.9% (8737/8748) of the reference genome transcripts. As expected, principal component analysis (PCA) of expression profiles showed clustering of replicates by life cycle stage (Fig. 3B). This analysis allowed us to determine global expression patterns of SIDER2-containing transcripts in the main developmental stages of the parasite and in response to stress factors known to trigger amastigote differentiation [34].

**Fig. 5:**
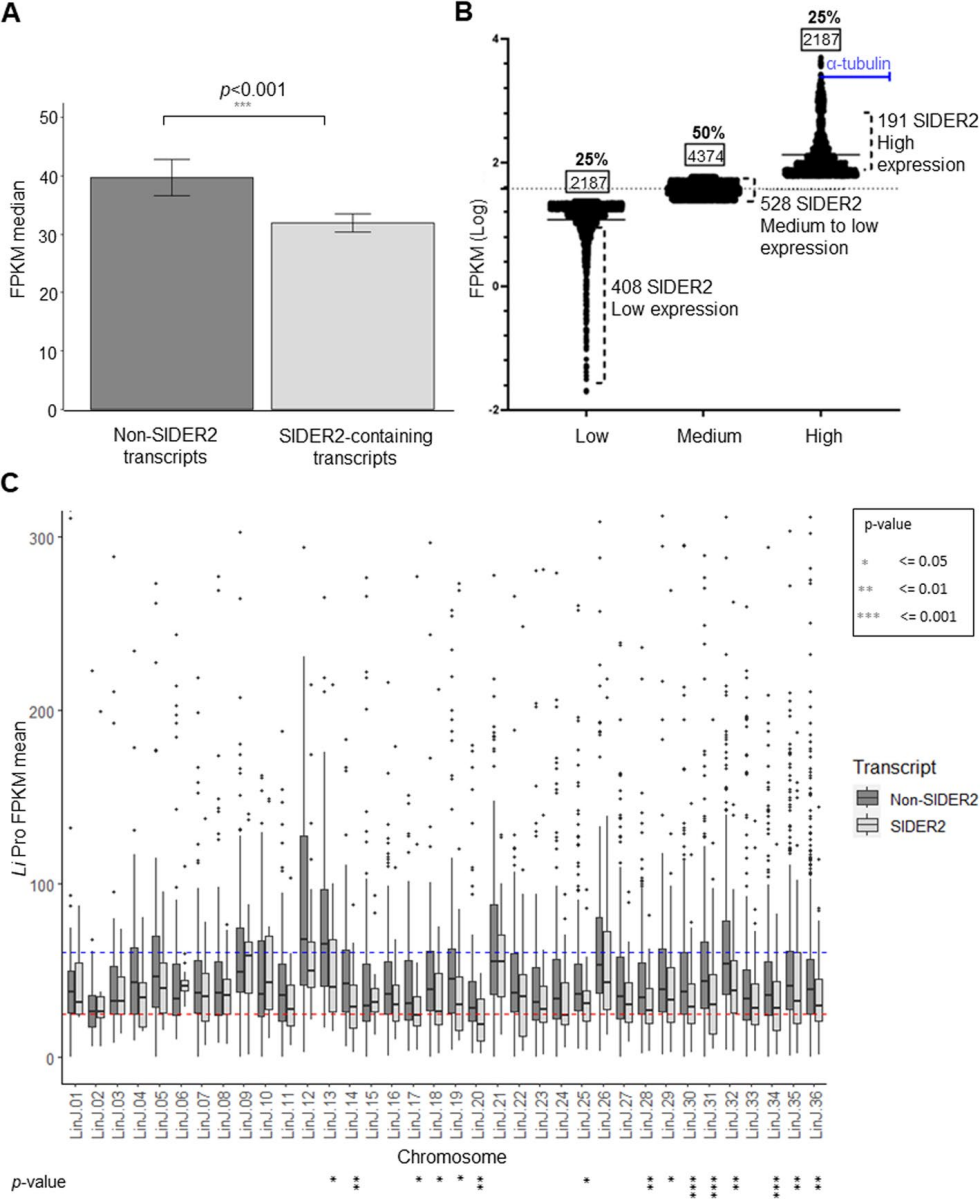
SIDER2-containing transcripts are generally less-expressed than non-SIDER2 transcripts. **A** Bar graph showing the significant difference in expression levels between SIDER2-containing and non-SIDER2 mRNAs, as determined by the Wilcoxon rank-sum test (*p* < 0.001). Fragments per kilobase of transcript per million mapped reads (FPKM) were used as readout of expression. **B** SIDER2-containing mRNAs were clustered using the interquartile range of expression levels at low (25% IQR), medium-to-low (50% IQR), and high (25%-IQR) expression based on FPKM values. Less than 15% of the SIDER2 transcripts exhibit high expression and the vast majority is below the average expression in the total *L. infantum* transcriptome. **C** Differential expression between SIDER2-containing and non-SIDER2 transcripts per chromosome. Boxplot of the expression levels in promastigotes for SIDER2-containing and non-SIDER2 transcripts across the 36 chromosomes of *L. infantum* JPCM5. The *y* axis was resized (0 to 300) to have a better resolution of the boxplots. The red and blue lines represent respectively the FPKM mean IQR25 and 75 (24.89, 60.50) used to define groups of low-, medium-, and high-expressed transcripts. Significance expression levels between SIDER2-containing and non-SIDER2 transcripts were evaluated for each chromosome using the Wilcoxon rank-sum test (*p < 0.05, **p < 0.01, ***p < 0.001)

Transcriptomic profiling of *L. infantum* promastigotes revealed that SIDER2-harboring transcripts were significantly generally less expressed than non-SIDER2 mRNAs (Wilcoxon rank-sum test; *p* < 0.001) (Fig. 5A and Additional file 1: Table S4). No substantial differences in expression between sense- and antisense-oriented SIDER2 elements were observed (Additional file 2: Fig. S5). We employed the interquartile range (IQR) of expression levels to further delineate expression patterns of SIDER2-containing mRNAs based on FPKM (*f*ragments *p*er *k*ilobase of transcript per *m*illion mapped reads) values. Consistent with this linear analysis, 408 SIDER2-bearing transcripts were clustered into the very low-to-low expression group (< 25% quartile), 528 into the medium-to-low category (< 50% quartile), and only a modest proportion of 191 SIDER2-transcripts (17%) was found within the high expression group (< 25% quartile) (Fig. 5B). Furthermore, we investigated whether the median expression levels of SIDER2-containing transcripts per chromosome were generally lower than that of non-SIDER2 transcripts. This analysis revealed that SIDER2-transcripts were significantly less expressed than non-SIDER2 mRNA in 15 chromosomes (Wilcoxon rank-sum test; *p* < 0.05) (Fig. 5C). Notably, Fig. 5C showed that the SIDER2-bearing transcripts were on average less expressed than the non-SIDER2 transcripts in most chromosomes (29 out of 36), except for chromosomes 2, 3, 6, 9, 10, 15, and 21 (see also Additional file 1: Table S5). Even for chromosomes 12, 13, 26, and 32 showing the highest overall expression in *L. infantum* promastigotes compared to the rest of the chromosomes, SIDER2-containing transcripts were expressed at lower levels than non-SIDER2 transcripts (Fig. 5C). Interestingly, most of the SIDER2 transcripts derived from aneuploid chromosomes (5, 12, 13, 26, and 31), except for chromosomes 9 and 21, present in three copies in the *L. infantum* JPCM5 strain used as determined by Illumina DNA-seq analysis (Additional file 2: Fig. S6), were less expressed than the median of non-SIDER2 transcripts (Fig. 5C and Additional file 1: Table S5).

**Fig. 6:**
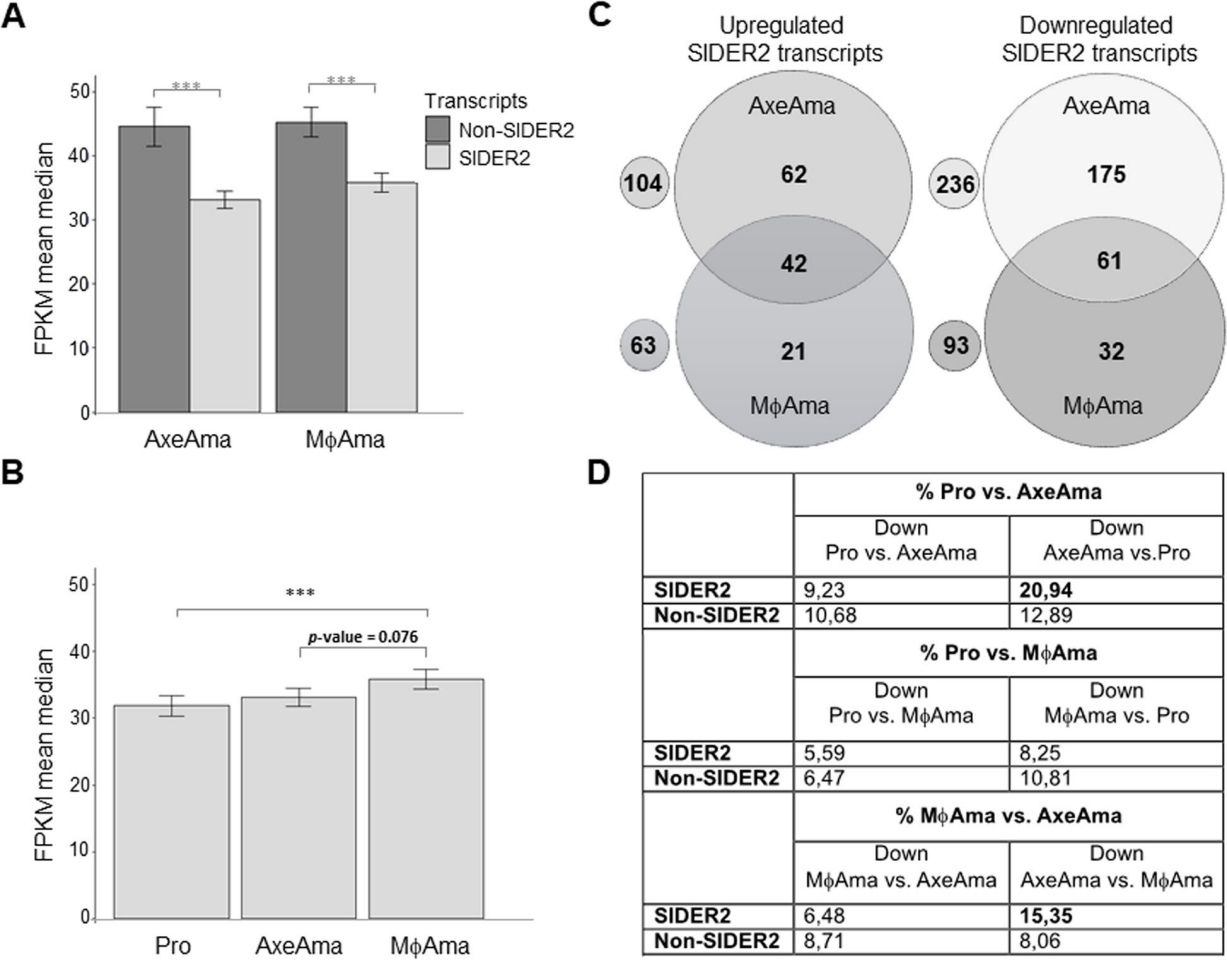
Developmental regulation of SIDER2-containing transcripts. **A** Comparative expression analysis between SIDER2-containing and non-SIDER2 transcripts in cultured axenic amastigotes (Axe Ama) and intracellular amastigotes derived from in vitro-infected macrophages (MϕAma). Significance levels were assessed using the Wilcoxon rank-sum test (*p < 0.05, **p < 0.01, ***p < 0.001). **B** Within the subset of SIDER2-containing transcripts, a notable difference in expression was observed between promastigotes (Pro) and MϕAma. **C** Numbers of SIDER2-containing transcripts upregulated in either axenic amastigotes or macrophage-derived amastigotes or shared by both (left panel). Numbers of SIDER2-containing transcripts downregulated in either axenic amastigotes or macrophage-derived amastigotes or shared by both (right panel). **D** Table summarizing the percentage of SIDER2-containing vs. non-SIDER2 transcripts that are developmentally regulated between *L. infantum* promastigote and amastigote life stages

Next, we investigated the genome-wide expression of SIDER2-bearing transcripts in the amastigote stage through comparative Illumina sequencing analysis of poly(A) + RNA between *L. infantum* macrophage-derived amastigotes or axenic amastigotes and promastigotes. SIDER2-containing mRNAs were generally less expressed than non-SIDER2 transcripts (FPKM median of 35.8 vs. 45) both in axenic and intracellular amastigotes (Fig. 6A), similar to what was seen for promastigotes (Fig. 5A, B). Notably, both in axenic amastigotes cultured under conditions triggering differentiation and in intracellular amastigotes ~ 62% (694 out of 1127) of SIDER2-bearing transcripts showed expression levels below the median (Additional file 1: Table S4). A significant difference in the expression of SIDER2-bearing transcripts was observed between promastigotes and macrophage-derived amastigotes but not between promastigotes and axenic amastigotes (Fig. 6B). Overall, high-throughput transcriptomic profiling highlights the association of SIDER2 elements with low-abundant mRNAs, which is consistent with our previous findings supporting that SIDER2s mediate mRNA decay (12, 28, 29, 33).

### Transcribed SIDER2 retroposons contribute to developmental gene expression

To assess whether SIDER2 elements were associated with stage-regulated gene expression, we compared expression levels of SIDER2-bearing transcripts between *L. infantum* promastigotes, conditions triggering amastigote differentiation (axenic amastigotes), and intracellular amastigotes extracted from in vitro-infected macrophages. This analysis revealed that there was a considerable proportion of SIDER2 transcripts downregulated (lowly expressed) in promastigotes but not in macrophage-derived or in axenic amastigotes (125 out of 1127; 11.1%) and others that behave in the opposite way (downregulated in amastigotes but not in promastigotes, 268 out of 1127; 23.8%). (Fig. 6C and Additional file 1: Tables S4 and S6). These differentially expressed SIDER2 transcripts (≥ 1.5-fold; log2 ± 0.58) account for ~ 35% of the SIDER2-containing transcripts when considering all the conditions analyzed. There was a trend for the developmentally regulated SIDER2-containing transcripts with a higher proportion being downregulated than upregulated in amastigotes, especially under conditions triggering amastigote differentiation (Fig. 6C, D). Specifically, 236 out of 1127 SIDER2 transcripts (20.9%) were downregulated under pH and temperature stress triggering amastigote differentiation (axenic amastigotes) compared to promastigotes vs. 104 (9.23%) that were upregulated under the same conditions (Fig. 6C, D). Similarly, but to a lesser extent, 93 (8,25%) of the SIDER2-containing transcripts were downregulated in macrophage-derived amastigotes compared to promastigotes vs. 63 (5.59%) being upregulated (Fig. 6C, D). It is worth mentioning that a considerable proportion of SIDER2-bearing transcripts downregulated in amastigotes codes for kinases/phosphatases and RNA-binding proteins (~ 1/3 of the transcripts) and several others for virulence factors, transporters, and metabolic genes (Additional file 1: Table S6).

To assess if there was an enrichment for the SIDER2 transcripts over the non-SIDER2 transcripts among the developmentally regulated transcripts across the *L. infantum* transcriptome, we calculated the percentage of SIDER2 vs. non-SIDER2 differentially expressed transcripts. For this analysis, we considered the proportion of SIDER2-containing (~ 13%; 1,127 transcripts) vs. non-SIDER2 transcripts (87%; 7,621 transcripts) in the *L. infantum* transcriptome. With a few exceptions, the percentage of SIDER2 vs. non-SIDER2 stage-regulated transcripts was generally comparable for several of the conditions tested, which suggests that proportionally SIDER2 elements contribute as much as non-SIDER2 sequences to developmental gene regulation (Fig. 6D and Additional file 1: Table S4). More specifically, 5.59% of the SIDER2 transcripts vs. 6.47% of non-SIDER2 transcripts were downregulated in promastigotes compared to macrophage-derived amastigotes while 8.25% of the SIDER2 transcripts vs. 10.81% of non-SIDER2 transcripts behave in the opposite way (Fig. 6D and Additional file 1: Table S4). Similarly, no enrichment was observed for SIDER2 (9.23%) vs. non-SIDER2 transcripts (10.68%) downregulated in promastigotes compared to axenic amastigotes (Fig. 6D and Additional file 1: Table S4). On the other hand, a significant enrichment was observed for SIDER2 transcripts (20.94%) over the non-SIDER2 transcripts (12.89%) downregulated in axenic amastigotes compared to promastigotes (Fig. 6D and Additional file 1: Table S4). In line with these results, SIDER2 transcripts downregulated in axenic amastigotes vs. macrophage-derived amastigotes were enriched (15.4%) over the non-SIDER2 transcripts (8.1%), suggesting that SIDER2-mediated degradation is triggered, but not only, by amastigote differentiation signals.

### Several transcripts encoding key Leishmania virulence factors harbor SIDER2 sequences as part of their 3'UTR

Our analysis revealed that several transcripts encoding well-characterized *Leishmania* virulence factors harbor SIDER2 retroposons in their 3'UTR that may regulate their expression. Most of these transcripts are developmentally regulated and have been associated with *Leishmania* infectivity or parasite viability in the mammalian host (Table 1. and Additional file 1: Table S6). Examples of SIDER2-bearing transcripts related to virulence exclusively or preferentially expressed in amastigotes include the following: cysteine proteases believed to degrade lysosomal proteases [35, 36], A2 proteins associated with visceralization [37, 38] and the response to stress [39, 40], members of the surface amastin protein family [16, 41], tryparedoxin peroxidase participating in the oxidative stress response [42, 43], 3'-nuclease/nucleotidase p1/S1 involved in purine salvage thought to be crucial for survival inside the phagolysosome [44, 45], glucose/SWEET transporters important for parasite virulence and infectivity [46, 47], argininosuccinate synthase regulating L-arginine metabolism essential for survival in the mammalian host [48], aminophospholipid translocase [49] and the choline transporter, components of the phospholipid and sphingolipid metabolism playing a pivotal role in the intramacrophage *Leishmania* proliferation [50], secretory lipase class3 participating in host lipid degradation and alteration of phagolysosome membrane [51], and the malonyl-Coa decarboxylase involved in fatty acid biosynthesis [52] (see Table 1. and Additional file 1: Table S6).

**Table 1:** Selected developmentally regulated SIDER2-containing transcripts encoding known *Leishmania *virulence factors

**SIDER2-containing transcripts encoding known** *Leishmania* **virulence factors**				
**TriTryp Gene ID**	**Putative function**	**Axe Ama**	**Mϕ** **Ama**	**Pro /** **Meta**
LINF_220012800	A2 protein	+	+	-
LINF_220012600, LINF_220012400,	A2-related	+	+	-
LINF_220014400				
LINF_220014500, LINF_220012300	3'a2rel-related protein	+	-	-
LINF_300020200	p1/s1 nuclease (3'-nucleotidase/nuclease)	+	+	-
LINF_330037600	cysteine peptidase - Clan CA - family C51 putative	+	+	-
LINF_160019700	OTU-like cysteine protease - putative	-	+	-
LINF_130006900	Lipase (class 3) - putative	+	+	-
LINF_230007900	argininosuccinate synthase – putative	+	+	-
LINF_360058200	Sugar efflux transporter (SWEET)	+	+	-
LINF_110012300	Protein Associated with Differentiation	+	+	-
LINF_340039100	aminophospholipid translocase – putative	+	+	-
LINF_140008800	Plasma-membrane choline transporter - putative	+	+	-
LINF_300028900	sphingosine 1-phosphate lyase	-	+	(+)
LINF_070013000	malonyl-coa decarboxylase-like protein	+	+	-
LINF_240017900, LINF_280019700	amastin-like surface protein-like protein	+	-	-
LINF_260013000	glutathione peroxidase-like / tryparedoxin peroxidase	+	-	-
LINF_020006900	phosphoglycan beta 1-3 galactosyltransferase	+	-	-
LINF_050017500	surface antigen-like protein	-	-	+
LINF_120013500	surface antigen protein 2 (PSA-2) - putative	-	-	+
LINF_340037800	lipophosphoglycan biosynthetic protein (lpg2)	-	-	+
LINF_240008400, LINF_240008300	UDP-galactose transporter - putative (LPG5A)	-	-	+
LINF_170015800	META domain containing protein (META2)	-	-	+
LINF_100013850, LINF_100013900	amino acid permease 24 - putative	-	-	+
LINF_340005600	ascorbate peroxidase	-	-	+
LINF_170007400	receptor-type adenylate cyclase	-	-	+
LINF_270016000, LINF_270016100	ATP-binding cassette subfamily A - member 1	-	-	+

In addition, there were SIDER2-containing transcripts encoding virulence factors preferentially expressed in promastigotes or infective metacyclics. These include *Leishmania* promastigote surface phosphoglycans like the lipophosphoglycan LPG2 contributing to parasite invasion, macrophage signaling, and survival within the host macrophage [53–56], as well as UDP-galactose transporters participating in phosphoglycan synthesis [57]; the surface antigen protein 2 (PSA-2) expressed predominantly in metacyclic promastigotes and playing a role in the resistance to complement lysis [58]; the amino acid permease 24 that transports proline/alanine [59] and plays a critical role in amino acid homeostasis and the response to osmotic shock [60]; the sphingosine 1-phosphate lyase that degrades sphingolipid (SL) metabolites (SLs represent 5–10% of *Leishmania* membrane lipids) shown to play an important role in metacyclogenesis and parasite virulence [61, 62]; the META protein upregulated in metacyclic promastigotes and associated with the response to oxidative and heat stress [63]; the ascorbate peroxidase shown to regulate intracellular H_2_O_2_ levels, metacyclogenesis, and survival within macrophages [64]; receptor adenylate cyclases on the parasite surface detecting environmental signals that affect the parasites’ responses to host immune challenge [65], and ABC transporters related to phospholipid trafficking contributing to infectivity [66] (Table 1. and Additional file 1: Table S6). Altogether, these data emphasize the association of SIDER2 sequences with several of the known key virulence factors in *Leishmania*. Our results do not, however, allow us to comment on a broader role of SIDER2s in regulating *Leishmania* virulence considering the limited knowledge on the subject to date.

### GO enrichment analysis reveals a functional bias for SIDER2-bearing transcripts

**Fig. 7:**
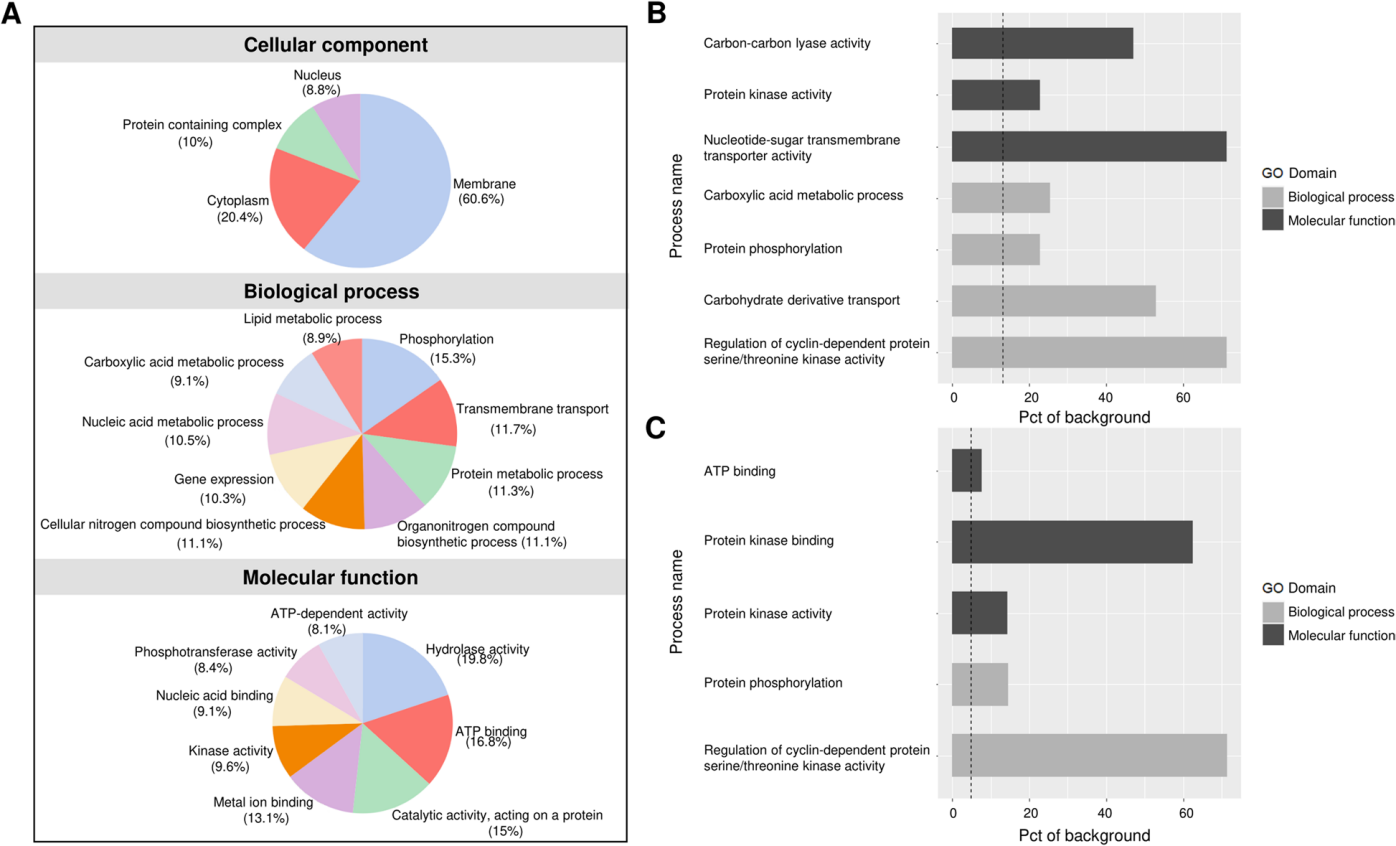
Gene Ontology (GO) enrichment analysis of *L. infantum* SIDER2-containing transcripts*.* From the 1127 SIDER2-containing mRNAs, a total of 799 (71%) had a related GO term. The OmicsBox tool (version 1.2) was used in this analysis (www.biobam.com/omicsbox). **A** Graphical representation of GO terms of SIDER2-containing transcripts in *L. infantum.* Pie graphs of the enriched GO terms were created for three categories: cellular component, biological process, and molecular function. **B** GO term enrichment in SIDER2-containing mRNAs. The vertical dash line marks the random frequency of a GO term for SIDER2-containing mRNAs (1127 SIDER2 transcripts/8527 total *L. infantum* transcripts; 13.2%). Pct (percentage) of background indicates the frequency at which a specific GO term is represented in the SIDER2ome. **C** GO term enrichment in the 408 SIDER2-bearing transcripts exhibiting the lowest expression. *t*-test considering adjusted *p*-value < 0.05 followed with Benjamini correction was applied using the OmicsBox tool. Only the more specific significant GO terms by process are represented here

To investigate whether some cellular functions were preferentially targeted for SIDER2-mediated regulation than others, we examined the distribution of Gene Ontology (GO) terms in proteins encoded by SIDER2-containing transcripts using two approaches. Initially, we submitted the FASTA sequences of the 1127 SIDER2-containing transcripts to the OmicsBox tool and obtained 799 with complete GO annotation (Additional file 2: Fig. S7). Subsequently, we submitted the feature Gene IDs to TriTrypDB, a specific database for trypanosomatids, and then used the Enrichment tool [67]. Our analysis revealed several processes significantly enriched in the SIDER2ome compared to the background genome (Benjamini corrected *p*-value ≤ 0.05) (Fig. 7A). GO analysis based on biological process indicated a SIDER2 enrichment in transcripts encoding proteins involved in phosphorylation (*p* = 0.03), regulation of cyclin-dependent protein kinase activity (*p* = 0.04), carboxylic acid metabolism (*p* = 0.03), and carbohydrate derivative transport (*p* = 0.03) (Fig. 7B and Additional file 1: Table S7). Furthermore, the molecular function analysis revealed an enrichment for transferase activity (*p* = 0.004), protein kinase activity (*p* = 0.01), carbon–carbon lyase activity (*p* = 0.046), and carbohydrate derivative transmembrane transporter activity (*p* = 0.004) (Fig. 7B and Additional file 1: Table S7).

Next, we investigated GO enrichment specifically in the SIDER2-containing transcript group exhibiting the lowest expression across the *L. infantum* genome (408 SIDER2 transcripts). The biological process GO analysis of these very low-expressed transcripts revealed an enrichment in protein phosphorylation (*p* < 0.0001) and regulation of cyclin-dependent protein serine/threonine kinase activity (*p* = 0.0001) (Fig. 7C and Additional file 1: Table S8), as also observed for the SIDER2ome (Fig. 7B). The molecular function analysis underscores a significant enrichment of low-expressed SIDER2-bearing transcripts in protein kinase activity (*p* < 0.0001), protein kinase binding (*p* = 0.0004) and ATP binding (*p* = 0.004) (Fig. 7C and Additional file 1: Table S8**)**. Although the overall analysis differs, there was also a relative enrichment of kinases among the low expressed non-SIDER2 transcripts. This is mainly because these transcripts represent a set of 2187 genes compared to 408 genes for the SIDER2 group. Among the 2187 low expressed genes, 86 are kinases with 30 belonging to the SIDER2 group (6.94%) and 56 to the non-SIDER2 group (3.19%). The resulting odds ratio of 2.18 (6.94/3.19) using the Fisher’s exact test (*p*-value 0.0177) suggests that the likelihood of a kinase being lowly expressed is 2.18 times higher in the SIDER2 group than in the non-SIDER2 group.

The GO enrichment analysis of developmentally regulated SIDER2-containing transcripts revealed interesting similarities and differences between intracellular and axenic amastigotes. Notably, for transcripts downregulated in axenic amastigotes, we found a significant enrichment for protein phosphorylation (biological process/BP; *p* < 0.003) and protein kinase activity (molecular function/MF; *p* < 0.001), transmembrane transport (BP; *p* < 0.01), transmembrane transporter activity (MF; *p* < 0.01), and integral membrane components (Cellular component; *p* < 0.001) (Additional file 1: Table S9). On the other hand, transcripts downregulated in macrophage-derived amastigotes were similarly enriched for protein phosphorylation (*p* < 0.006) and protein kinase activity (*p* < 0.01) but not for transmembrane transport and transmembrane transporter activity (Additional file 1: Table S9). Altogether, this analysis provides valuable insights into the functional categories encoded by different categories of SIDER2-bearing transcripts.

## Discussion

Short interspersed degenerated retroposons (SIDERs) in *Leishmania* represent the largest family of repetitive elements among trypanosomatids. The substantial expansion of SIDER sequences across *Leishmania* genomes provides compelling evidence that these formerly active retroposons have endured remarkable domestication by their host genomes and most likely helped the parasite gaining an auspicious evolutionary edge in its complex lifestyle. This study provides a holistic view of important features of SIDER2 retroposons with novel insights into their high evolutionary divergence, chromosomal organization, and clustering and major role in monitoring mRNA abundance throughout the parasite development. The high throughput genome and transcriptome analysis of the *L. infantum* “SIDER2ome” represents an important step forward in our understanding of how these widespread repetitive elements shape genome evolution and influence gene expression.

Even though SIDER2 sequences are uniformly scattered throughout the *Leishmania* genome, as also reported previously [12, 26, 31], their integration is not random. Remarkably, more than 87% of *L. infantum* SIDER2 elements were found in 3'UTRs [12, 26, 31], supporting their primary function as major cis-regulatory elements. Genome-wide surveys in other eukaryotes revealed transposable elements (TEs) as a substantial source of cis-regulatory elements in diverse eukaryotic species [68, 69]. The best documented are Alu elements in human inserted within 3ʹUTRs providing recognition sequences for Staufen 1-mediated mRNA decay [70]. It is worth mentioning that 3'UTRs harboring SIDER2 elements are on average 3 times longer than those of non-SIDER2 transcripts and that the least abundant SIDER2 transcripts have the longest 3'UTRs. This suggests that SIDER2-bearing transcripts have a potential for higher and more complex posttranscriptional regulation. In addition to their predominant location in 3'UTRs, SIDER2 sequences were found in strand-switch regions where polycistronic transcription is initiated or ended. The 5.5-fold enrichment of SIDER2 sequences in divergent vs. convergent strand-switch regions along with the lack of typical promoters in these parasites [71–73] suggests a possible role in supporting transcription initiation. Interestingly, the conserved 79-nt signature from the distantly related L1Tc-retroposon in *T. cruzi* was shown to promote transcription [74] and increasing evidence in mammalian cells indicates that retrotransposon-derived sequences can act as enhancers and promoters [68, 75].

Notwithstanding the common ancestry of SIDER2 elements, they were distanced through evolution due to differential selection pressure to form various distinct groups. In fact, our comprehensive analysis revealed a high evolutionary divergence between SIDER2 sequences with 311 distinct SIDER2 clusters identified across the 36 *Leishmania* chromosomes. The high SIDER2 phylogenetic distribution suggests their relatively ancient origins. A fascinating finding here is the chromosomal organization of SIDER2 elements. In fact, 88.4% of the SIDER2 clusters harbor sequences that are usually located nearby on a chromosome. Interestingly, the closer the SIDER2 sequences are positioned on the chromosome, the higher is their homology. SIDER2 chromosomal proximity relationship has also been reported previously on *L. infantum* chromosomes 20 and 32 [76]. The presence of homologous SIDER2 copies nearby in the linear sequence of chromosomes might be the result of ancient insertions by the typical copy-and-paste mechanism (replicative transposition) of class-I TEs (retroposons) [77]. This possibility is supported by our data demonstrating that the vast majority of homologous SIDER2 sequences within each cluster are not the result of paralog/segmental gene duplication events. This intriguing chromosomal organization of homologous SIDER2 elements is thought to be an evolutionary adaptation to environmental challenges which may favor chromosome dynamics and or co-regulation, among other functions. Accordingly, we have detected several clusters where most of the SIDER2-containing transcripts display similar levels of expression, which suggests they can be co-regulated by SIDER2 sequences sharing similar regulatory properties. The percentage of putatively co-regulated SIDER2 clusters in the different life stages analyzed was not that high (~ 14%), but it is likely that this number can vary depending on the many rapidly changing environments the parasite encounters over time throughout its development in the insect vector and the mammalian host. There is no experimental evidence yet supporting that SIDER2 clusters can form post-transcriptional regulons. Nonetheless, it is theoretically possible that SIDER2s exert a more extensive influence in regulatory evolution by coordinating the expression of multiple gene products that function in concert to control entire pathways and complex biological processes. Post-transcriptional regulons have been described previously in the related parasite *T. brucei* [78–80]. Moreover, the correlation between SIDER2 chromosomal proximity and sequence similarity can favor genomic rearrangements resulting in the amplification of specific loci as a mechanism of genomic adaptation to a changing environment altering gene expression. This may be a way for the parasite to increase gene expression levels, when needed, considering that SIDER2 elements often contribute to low expression. Our previous studies have emphasized the implication of SIDER2 sequences in stochastic genomic rearrangements as a stress adaptation strategy in *Leishmania* [81]. SIDER elements account for 68% of repetitive elements in *Leishmania*, hence playing a major role in genome evolution and plasticity.

This study established the first SIDER2 transcriptomic profiling in the main developmental stages of *Leishmania*, as well as in culture conditions triggering promastigote to amastigote differentiation. This is highly significant given that SIDER2-contaning transcripts account for ~ 13% of the *L. infantum* transcriptome. A central finding in this study is that SIDER2 elements are overrepresented in lowly expressed mRNAs. We found that SIDER2-contaning transcripts were generally less expressed than non-SIDER2 transcripts in all growth conditions and developmental stages tested. The link of SIDER2 elements with low levels of expression is generally independent of their chromosomal location, length, or orientation and suggests a broad contribution of these sequences in monitoring mRNA abundance during the parasite development. These findings corroborate our previous work on a few selected SIDER2s in *L. major* demonstrating that SIDER2-containing transcripts undergo rapid decay and that SIDER2 elements were responsible for degradation initiated through endonucleolytic cleavage (12, 28, 29, 33). It is remarkable that despite their high length and sequence diversity, most SIDER2 sequences were associated with low mRNA expression. This implies that various SIDER2 sequences or structures engage the binding of different trans-acting factors contributing either to mRNA degradation or decreased accumulation via distinct regulatory networks. We have previously identified members of the Pumilio protein family as players contributing to SIDER2-mediated mRNA decay [82]. Low-abundant SIDER2-containing transcripts can be the result of rapid decay as it was shown before [28, 33], but we cannot exclude that for some transcripts, their notable instability is due to reduced translation efficiency. The fact that ~ 70% of SIDER2 elements share different degrees of sequence similarity with the hallmark 79-nt signature II sequence, shown previously to be essential for mRNA degradation [28, 33], attributes a central role of this sequence in the regulation of mRNA abundance. The SII sequence conservation through evolution is a strong predictor of biological function and further supports SIDER2’s co-option as regulatory elements. There is also a smaller percentage of SIDER2 sequences that are associated with high levels of expression under the conditions analyzed, suggesting that this SIDER2 group may fulfill different regulatory functions.

SIDER2 sequences add to the diversification of the *Leishmania* gene expression repertoire in response to different environmental stimuli. Here, we show that a significant proportion of SIDER2 transcripts (~ 35%) were differentially regulated depending on the parasite developmental stage, the intricate environment of the macrophage phagolysosome, or the exposure to factors triggering promastigote to amastigote differentiation. SIDER2 sequences could contribute to developmental gene expression by downregulating mRNA expression in one stage but not, or not to the same extent, in the other. Our previous observations support that SIDER2-mediated regulation can be modulated by external factors specific to each developmental stage of the parasite. Studies on selected developmentally regulated SIDER2-containing transcripts [10] demonstrated that SIDER2 sequences were able to promote rapid mRNA turnover in promastigotes but not in amastigotes [29]. The mechanisms behind are not yet well understood but could be attributed to the differential expression of trans-acting factors (or ncRNAs) bound SIDER2 sequences or to the differential affinity of these factors due to changes in RNA structure that could block or decrease degradation. Changes in temperature or pH may alter SIDER2 RNA conformation facilitating or preventing binding of trans-acting factors. Given the great diversity of SIDER2 sequences (and possibly structures), we cannot exclude that some SIDER2 elements do not degrade their target RNAs but use other modes to regulate them in a stage-specific manner. Further investigation may shed light on the different modes of regulation by SIDER2 elements during the parasite development. Interestingly, we found that several of the developmentally regulated transcripts encoding known *Leishmania* virulence factors associated with infectivity, or viability in the mammalian host, or resistance to the host immune response harbor SIDER2 sequences in their 3ʹUTR, which suggests that SIDER2s are likely to play a pivotal role in their regulation. These include, but not only, major *Leishmania* surface membrane components like LPG2, the promastigote antigen protein 2 and members of the amastin glycoprotein gene family, transmembrane carbohydrate and lipid transporters, and stress-related factors. LPG2 (lipophosphoglycan 2) is a Golgi GDP-mannose transporter involved in the biosynthesis of phosphoglycans [83] present on LPG and other surface or secreted molecules that has been shown to play critical roles in the parasite invasion, macrophage signaling and intracellular trafficking, and the host early immune response [53–56]. The promastigote antigen protein 2 protects the parasite from lysis by the complement [58] and amastins play a role in parasite–host interaction and the viability of intracellular amastigotes [84]. Glucose/galactose transporters ensuring the availability of different carbon sources in the macrophage phagolysosome [46, 47] and components of the phospholipid and sphingolipid metabolism important for metacyclogenesis and survival in the mammalian host [50, 61, 62] are also possibly regulated by SIDER2. In addition, there are several stress-related factors, such as the A2 and A2-related proteins [39, 40], tryparedoxin peroxidase [42, 43], and the META cluster [63] involved in the response to heat or oxidative stress which are subject to SIDER2-mediated regulation. At this stage, we cannot conclude on a much broader role of SIDER2 elements in the parasite virulence. The proportion of SIDER2 transcripts encoding virulence factors vs. those which are unrelated to virulence cannot currently be established because we do not have functional data for the vast majority of SIDER2-containing transcripts. Besides, many transcripts harboring SIDER2 encode hypothetical proteins, which complicates functional analysis and limits predictions on their putative role in virulence or pathogenesis.

Interestingly, our data support some functional bias for SIDER2-bearing transcripts. We found that SIDER2s were generally enriched in transcripts encoding integral components of the parasite membrane, transmembrane carbohydrate and lipid transporters, as well as proteins involved in phosphorylation, kinase binding, and regulation of kinase activity. Dynamic processes, like import of nutrients and signal transduction pathways, are in accordance with the rapidly changing environments the parasite encounters throughout its development. *Leishmania*, acquire a plethora of nutrients from their hosts, employing transport proteins located in the plasma membrane of the parasite [85, 86]. Sugars and their derivatives are tightly linked to *Leishmania* amastigote differentiation and regulating their transport and availability are crucial to control growth inside the macrophage phagolysosome [87]. GO pattern enrichment of SIDER2-bearing transcripts may differ depending on their expression levels during the parasite intracellular development. For example, while SIDER2 transcripts downregulated in intracellular amastigotes were enriched for protein phosphorylation and kinase activity, those upregulated were enriched for transmembrane transport and transmembrane transporter activity.

In summary, this comprehensive characterization of SIDER2 retroposons has largely expanded our knowledge of the outstanding features of these repetitive elements, representing the largest retroposon family in trypanosomatids. These findings provide important insights into evolutionary innovations of SIDER2 retroposons with respect to their chromosomal organization and clustering likely to favor genome dynamics and co-regulation, as well their critical role as major regulators of mRNA abundance during the parasite development. A major outstanding task still is to gain a better grasp of the role of SIDER2 co-option in driving the evolution of *Leishmania* gene regulatory networks on a broader scale and of how these processes link to pathogenesis.

## Conclusions

This study provides important conceptual advances into evolutionary innovations of transcribed extinct retroposons in *Leishmania* representing the largest family of repetitive elements among trypanosomatids. Studies at the genome-wide level have shown that short interspersed degenerated retroposons of the SIDER2 subfamily are mostly transcribed as part of 3'UTRs and are over-represented in lowly expressed transcripts. The remarkable link of SIDER2 retroposons with downregulation of gene expression supports their co-option as major cis-regulators of mRNA abundance in *Leishmania*. SIDER2 sequences also add to the diversification of the *Leishmania* gene expression repertoire with several SIDER2-containing transcripts being differentially regulated during the parasite development. Another important finding here is that SIDER2 elements form various evolutionary divergent clusters, each harboring homologous SIDER2 sequences usually located nearby on the chromosome. This intriguing chromosomal organization of homologous SIDER2 elements thought to be an evolutionary adaptation to environmental challenges underscores the importance of SIDER2 proximity in shaping chromosome dynamics and co-regulation. Overall, these findings contribute significantly to our understanding of how these widespread repetitive elements shape genome evolution and influence gene expression.

## Methods

### Parasite culture and macrophage infection

*Leishmania infantum (*MHOM/MA/67/ITMAP‐263) promastigotes were cultured in SDM‐79 medium supplemented with 10% heat‐inactivated fetal calf serum (FCS) (Multicell Wisent Inc., Canada) and 5 µg/ml hemin at 25 °C and pH 7.0. Differentiation of *L. infantum* promastigotes into amastigote-like forms was induced by growing 1 × 10^6^ log-phase promastigotes in MAA/20 medium [88] in 25 cm^2^ flasks at 37 °C in the presence of 5% CO_2_ for three sub-passages prior to RNA extraction. Macrophage-derived amastigotes were collected from the human leukemia monocyte cell line (THP-1 cells) infected with *L. infantum* stationary promastigotes at a parasite/macrophage ratio of 15:1 as described previously [89]. After 2 h post-infection, non-internalized parasites were visually inspected and removed by several washes using 2% heat-inactivated horse serum (Gibco). Infected macrophages were incubated for 5 days prior to amastigote RNA extraction.

### Oligo (dT) mRNA capture and library preparation

*Leishmania infantum* log-phase promastigotes (5 × 10^7^ parasites/ml), passage 3 day 3 axenic amastigotes (5 × 10^7^ parasites/ml), and day 5 macrophage-infected intracellular amastigotes were harvested in Trizol (Invitrogen) and total RNA was purified using RNeasy® Plus Mini Kit (Qiagen, SAS, France) from three independent cultures. Oligo (dT) based enrichment (New England Biolabs) was used for mRNA purification. We used 6 µg of total RNA and processed it according to the instruction manual. The RNA quality was assessed by Bioanalyzer chips (Agilent) before and after mRNA capture.

### Illumina DNA and RNA-sequencing

The RNA-seq paired-end libraries were prepared using the Illumina® TruSeq® RNA Sample Preparation protocol, according to manufacturer’s instruction, in triplicate for each developmental stage of the parasite. Subsequently, libraries were sequenced in two different paired-end 100 pb sequencing runs on a NovaSeq 6000 to reach more than 50 million reads at the next-generation sequencing platform at the Genomics Center of the CHU de Québec-Université Laval Research Center, QC, Canada. Both sequencing runs were pooled, trimmed using Trimmomatic (v0.39) [90], and aligned to the *Leishmania infantum* JPCM5 genome V62 (https://tritrypdb.org/tritrypdb/app/downloads) [67] using the software HISAT2 (v2.2.1) [91]. The Illumina RNA-Seq aligning metrics were evaluated using the picard software (v 2.26.3) (http://broadinstitute.github.io/picard/). The software StringTie (v2.1.3) [92] was used to predict the expression levels from the HISAT alignment using the *L. infantum* JPCM5 V64 transcriptome available from https://tritrypdb.org/tritrypdb/app/downloads. *F*ragments *p*er *k*ilobase of transcript per *m*illion mapped reads (FPKM) values were used as a readout of expression levels. FPKM normalizes read count based on gene length and the total number of mapped reads. Differential gene expression between *L. infantum* promastigotes and amastigotes was analyzed using the DESeq2 software with multiple-testing correction [93]. Genes were considered as modulated (up or downregulated) when absolute fold changes were ≥ 1.5 and the false discovery rate (FDR)-corrected *p*-value was lower than 0.05 (*p*adj). The DNA-Seq library was prepared with the NEBNext Ultra II DNA library prep kit for Illumina (New Englands Biolabs Inc., Ipswich, MA, USA) according to manufacturer’s instruction and sequenced on a NovaSeq 6000 in paired-end 100 pb sequencing. Reads were trimmed by Trimmomatic (v0.39) and aligned to the *Leishmania infantum* JPCM5 genome V62 with Bowtie 2 (v2.5.1) [94]. To determine chromosome ploidy of the *L. infantum* JPCM5 reference strain used for RNA-Seq, the chromosome sequencing depth was evaluated using the samtools option “depth” (v1.17). The median sequencing depth by chromosome was calculated and normalized using the overall sequencing depth using R (v4.3.3). Reads count per gene were computed from the alignment file using HTSeq [95] and then converted to FPKM values by correcting for the total number of mapped reads and for gene length.

### SIDER2 sequence alignments and genomic distribution

Initial multiple alignments of SIDER2 sequences were obtained as previously reported by Smith et al. 2009 [26]. Our bioinformatics pipeline consisted of using a core consensus SIDER2 sequence encoded in a profile hidden Markov model (HMM) implemented in the HMMER-v.3.3.2 (26 Nov 2020) software (hmmer-3.3.2.tar.gz) (HMMER implements methods using probabilistic models called profile HMMs) to identify SIDER2 homologs and fragments across a recent version of the *L. infantum* genome (TriTrypDB-62_LinfantumJPCM5) and to perform multiple sequence alignments. Genomic coordinates from HMM hits were input in TriTrypDB (https://tritrypdb.org/) to further extract the SIDER2-FASTA sequences [67]. The prediction of polyadenylation and trans-splicing sites was first performed using the PRED-A-TERM algorithm [32]. Reads were aligned to the reference genome and visualized in Integrative Genomics Viewer (IGV_2.15.2®). Manual verification of each SIDER2 to their respective genomic position was also performed. Conservation of the hallmark 79-nt Signature II (SII) sequence among SIDER2s was evaluated with the bit-score using the *nhmmer* program implemented in HMMER software (v.3.3.2) against the SII consensus. We opted for *nhmmer* tool that utilizes a probabilistic model called profile HMM. Due to its powerful nucleotide alignment optimization algorithm, this tool is more sensitive than sequence-based methods [96–98]. Moreover, its previous version (i.e. v.1.8.5) has been successfully used to annotate transposable elements (TEs) in other studies [26, 99]. HMMER bit-score is a log2-odds ratio score that compares the likelihood of the profile HMM to the likelihood of a null hypothesis (as in BLAST). We created five HMMER groups based on the bit-score of each SIDER2 element with the SII. The maximum bit-score corresponding to a perfect match with SII was 66.5. The thresholds for each HMMER group were chosen by analyzing the SII alignment across SIDER elements using *hmmalign* and represented by JALVIEW (see Additional file 2: Fig. S1B). The HMMER groups 1 to 2 showed the highest SII conservation, while we began to observe missing parts and mismatches within the SII for the HMMER group 3, and ultimately, the HMMER 4 showed the less SII conservation across all sequence.

### SIDER2 sequence clustering and phylogenetic trees

SIDER2 nucleotide sequences were clustered by CD-Hit-Est (v.4.8.1) at 85% of identity with an alignment coverage of 10% (option: -c and -aL, respectively) [100]. Shared regions between SIDER2 with ≥ 80% of identity were calculated using the percentage of query coverage per subject (-outfmt “qcovs”) of BLASTN (v.2.9.0 + ; option: -task “blastn” -perc_identity 80 -outfmt “6 std qlen slen qcovs”) [101]. To generate the phylogenetic tree, SIDER2 sequences were aligned using MAFFT multiple sequence alignment program (v.7.471; option: –localpair –maxiterate 1000) [102]. Multiple alignments were used to compute the unrooted maximum likelihood tree using IQ-TREE (v.2.1.2; bootstraps: 1000; model: GTR + I + G1) [103]. Phylogenetic trees were visualized with FigTree (v.1.4.4). Heatmap functions and statistical analysis to normalize input data, run clustering algorithm, and visualize the result with dendrograms were generated in RStudio [104] (https://www.rstudio.com). A heatmap is a graphical representation of data that displays the relative intensity of values in a matrix as colors (https://www.data-to-viz.com/graph/heatmap.html). Paralogous genes within SIDER2 clusters were identified among the 1127 SIDER2-containing transcripts using the CD-Hit (v.4.8.1) software developing for protein sequences with ≥ 85% similarity over ≥ 90% of the sequence length.

### Gene ontology (GO) enrichment analysis

The FASTA sequences of the ORFs within a total of 1127 SIDER2-containing transcripts were exported from TriTrypDB (https://tritrypdb.org/tritrypdb/app) and imported into OmicsBox version 1.2 (BioBam) (www.biobam.com/omicsbox). Coding regions were aligned to the NCBI database using BLASTX search (E-value ≤ 1.0 × 10^–3^). Subsequent GO mapping was performed using the Blast2GO mapping against the latest version of the GO database to obtain the functional labels. Sequences that shared similarities with known proteins in BLASTX searches with significant similarity (E < le^−10^) were identified using the online tool InterProScan 5.0. Next, the appropriate GO term was allocated to its respective predicted function using an e-value cut-off of 1.0 × 10^–6^ and an annotation cut-off of 55 evidence code set to 0.8 for the different categories as implemented in OmicsBox. This analysis revealed 799 (70.9%) genes with complete GO annotation. Pie graphs of the enriched GO terms were created for three categories: biological process, cellular component, and molecular function for the SIDER2-containing transcripts and their subclusters. GO enrichment analysis was conducted using either Omics Box or the TryTripDB enrichment tool.

### Statistical analysis

Quantitative analysis was performed using Stata/SE® 13.1 for Windows (College Station, Texas, USA). The normality of FPKM values was attested by the Shapiro–Wilk test and Student’s *t*-test was used to compare means between SIDER2 and non-SIDER2 groups of the normalized values and the Wilcoxon test to compare medians of the non-normal distributions. Interquartile range (IQR) 25% and 75% was used to define groups of low and high expression, respectively. Values were logarithmically converted and normalized to generate diagrams.

## Supplementary Information

Additional file 1: Tables S1-S9. Table S1. A metadata table including the complete list of 1448 SIDER2 elements in *L. infantum* with 1262 present in 3'UTRs (predicted by PRED-A-TERM and confirmed for several by Illumina RNA-seq). This table also includes information regarding the genomic location and orientation of SIDER2 elements, their length, their distance from the CDS, CD-Hit-Est clustering, HMMER signature II score and the expression group in *L. infantum* promastigotes as determined by Illumina RNA-sequencing. Table S2. Metadata table of 1262 SIDER2 sequences located at the 3'UTR subjected to of CD-Hit-Est clustering analysis based on sequence similarity. In a separate sheet (≥ 4 SIDER2/cluster-no paralogs), the list of 74 clusters with ≥ 4 transcripts each harboring a single SIDER2 in their 3'UTR, excluding all paralogous proteins is provided. In another separate sheet (SIDER2 Clusters with paralogs), 19 clusters containing paralogous genes (among the 1127 *L. infantum* transcripts) harboring a single SIDER2 element in their 3'UTR are listed. In the last sheet (FPKM mean-SD Pro and Ama), expression levels (mean of FPKM values) of 74 clusters harboring up to 4 SIDER2-containing transcripts in *L. infantum* promastigotes (Pro), axenic amastigotes (Axe Ama) and macrophage-derived amastigotes (Macro Ama) are shown. Table S3. A list of 1127 *L. infantum* transcripts harboring a SIDER2 element in their 3'UTR as predicted by the PRED-A-TERM software. We also manually examined the Illumina RNA-seq read patterns in IGV to check if they were aligned with the 3' UTRs harboring SIDER2 elements. Table S4. A full list of the *L. infantum* transcriptome (FPKM values) in promastigotes (Pro), axenic amastigotes (Axe Ama) and macrophage-derived amastigotes (Macro Ama) as resulted from high throughput Illumina-RNA seq analysis. In a separate sheet, only the SIDER2-containing transcripts were analyzed. In another sheet, we summarized the proportion of developmentally regulated SIDER2-containing vs. non-SIDER2 transcripts (see also Fig. 6D). Table S5. Distribution of SIDER2 elements and expression levels of SIDER2-containing transcripts across the 36 *L. infantum* chromosomes. The comparison of the low-expressed SIDER2-containing transcripts to the low-expressed non-SIDER2 transcripts per chromosome is shown. The chromosome median expression is normalized by the number of chromosomal copies (as determined by Illumina DNA-seq). Table S6. List of developmentally regulated SIDER2-containing transcripts. SIDER2-containing transcripts preferentially expressed in *L. infantum* promastigotes (Pro) or axenic amastigotes (Axe Ama) and or macrophage-derived amastigotes (Macro Ama) are shown. The cutoff for differentially expressed (DE) SIDER2 transcripts was set at log2: 0.58. In separate sheets, stage-regulated SIDER2 transcripts similarly expressed in axenic and macrophage-derived amastigotes or solely in macrophage amastigotes are shown. Table S7. Table with *p*-values comparing Gene Ontology (GO) functions (e.g., Biological process/BP, Molecular function/MF and Cellular component/CC) of SIDER2-containing transcripts in *L. infantum*. Highlighted in red are GOs with a Benjamini corrected *p*-value < 0.05. Table S8. Table with *p-*values comparing GO functions (e.g., Biological process/BP, Molecular function/MF and Cellular component/CC) of low-expressed SIDER2-containing transcripts (408) in *L. infantum*. Highlighted in red are GOs with a Benjamini corrected *p*-value < 0.05. Table S9. Table with *p*-values comparing GO function’s enrichment (e.g., Biological process/BP, Molecular function/MF and Cellular component/CC) of developmentally regulated SIDER2-containing transcripts, up- or down-regulated either in *L. infantum* axenic amastigotes (AxeAma) or macrophage-derived amastigotes (MacroAma). Highlighted in red are GOs with a Benjamini corrected *p*-value < 0.05.

Additional file 2: Figs. S1-S7. Fig. S1. Nucleotide composition of SIDER2 consensus and their hallmark 79-nt signature II sequence. Jalview alignment was performed on the SIDER2 consensus sequence encompassing 536 bp. Within sense SIDER2s, a conserved 79-nt signature (Signature II/SII) resides at the 5'-end, while an A-rich tail typically at the 3'-end (A *left)*. The antisense SIDER2 is represented as the reverse complement of the sense element, featuring a T-rich stretch at the 3'-end and the SII at the 5'-end (A *right)*. B-C Multiple sequence alignments of SIDER2 sequences to the SII consensus using *hmmalign* and represented by JALVIEW. Signature II consensus or its reverse complement (RC) aligned sequences are shown. The black consensus bars below the alignments show the frequency of the most conserved base indicated underneath. Five SIDER2 sequences within each HMMER group (Groups 1-5) (B) and 25 antisense SIDER2 sequences (C) shown here were randomly selected. Fig. S2. Phylogenetic and CD-Hit-Est analyses of *L. infantum* SIDER2 sequences. A The unrooted maximum likelihood phylogenetic tree was made using IQ-TREE (v.2.1.2) model GTR + I + G1 of 1448 SIDER2 sequences aligned by MAFFT (v.7.471). SIDER2 sequences were colored by the chromosome where they are located. Three sections were enlarged for better resolution. Tips in the enlarged boxes were named by chromosome followed by the start position of SIDER2. B Concordance between the phylogenetic and CD-Hit-Est analyses. An unrooted maximum likelihood tree generated as indicated in (A) depicted 189 SIDER2 sequences from five chromosomes (LinJ.14, LinJ.16, LinJ.20, LinJ.22 and LinJ.27) aligned by MAFFT (v.7.471). Tips on the tree were color-coded according to their respective chromosomes. Each SIDER2 element is labeled with its corresponding CD-Hit-Est cluster (see Methods). The same tree is represented in Fig. 2B, but with the tips labeled by the LinJ transcripts harboring SIDER2 elements. Fig. S3. Heatmap of regions with ≥ 80% of sequence identity shared between 189 SIDER2 sequences from five *L. infantum* chromosomes. SIDER2s from chromosomes 14, 16, 20, 22 and 27 were organized based on their genomic position and aligned using BLASTn (-task "blastn" -% identity 80, V.2.9.0 +). The percentage of query coverage per subject with ≥ 80% of identity was calculated by the outfmt option "qcovs". The darker is the blue color, the higher is the query coverage. Dashed gray lines indicate the strand-switch regions. Heatmap of 189 SIDER2 sequences showed that i) SIDER2 sequences on the same chromosome are more homologous to each other than between SIDER2s present on different chromosomes and ii) within the same chromosome, SIDER2 sequences can be part of different clusters which do not necessarily share regions of homology. Fig. S4. SIDER2-bearing transcripts have generally much longer 3'UTRs than non-SIDER2 transcripts. The length of 3'UTRs predicted by PRED-A-TERM was compared between SIDER2-containing (N = 1127; 409 low, 527 medium-to-low, and 191 high expressed) and non-SIDER2 transcripts (N = 7207; 1540 low, 3726 medium, and 1941 high expressed). All SIDER2-bearing transcripts have significantly longer (> 3 times) 3'UTRs than non-SIDER2 transcripts, regardless of their expression levels. The statistical significance of these observations was assessed using the Kruskal-Wallis test. Fig. S5. Expression levels of transcripts harboring sense vs. antisense SIDER2 sequences. Bar graph representing the expression levels in *L. infantum* (*Li*) promastigotes (Pro) of sense (*n* = 855) and antisense (*n* = 153) SIDER2-containing transcripts. Only the expression of SIDER2-containing transcripts harboring a single SIDER2 element within their 3’UTRs was analyzed to avoid using the expression of the same gene more than once. Significance level was assessed using the Wilcoxon rank-sum test and resulted by *p-value* = 0.5588. Fig. S6. Comparative genomic sequencing vs. RNA-sequencing analysis illustrates that SIDER2-containing mRNAs are largely less expressed than non-SIDER2 transcripts. The dot chart illustrates FPKM (Fragments Per Kilobase of transcript per Million mapped reads) levels obtained from Illumina DNA sequencing (represented in A) and Illumina RNA sequencing in B. SIDER2-containing mRNAs are denoted in red, while non-SIDER mRNAs are represented in grey. The green line represents the mean, and the blue line shows the median of mRNA expression levels. Notably, most of the SIDER2-containing mRNAs exhibit expression levels below the median expression of the *L. infantum* transcriptome. Fig. S7. Schematic representation of the different steps used in GO analysis. A total of 1127 SIDER2-containing transcripts was included in GO analysis. A The FASTA sequences of the ORFs were exported from TriTrypDB (https://tritrypdb.org/tritrypdb/app) and imported into OmicsBox version 1.2 (BioBam) (www.biobam.com/omicsbox). Coding regions were aligned to the NCBI database using BLASTX search (E-value ≤ 1.0 × 10-3). Subsequent GO mapping was performed using the Blast2GO mapping against the latest version of the GO database to obtain the functional labels. Sequences that shared similarities with known proteins in BLASTX searches with significant similarity (E < le-10) were identified using the online tool InterProScan 5.0. Next, the appropriate GO term was allocated to its respective predicted function using an e-value cut-off of 1.0 × 10-6 and an annotation cut-off of 55 evidence code set to 0.8 for the different categories as implemented in OmicsBox. B Final analysis found 799 (70.9%) genes with complete GO annotation. Pie graphs of the enriched GO terms were created for three categories: biological process, cellular component, and molecular function for the SIDER2-containing transcripts and their subclusters.

## Data Availability

The DNA-seq and RNA-seq datasets generated using Illumina deep sequencing have been deposited in the NCBI Bioproject database under accession number PRJNA1071080, (https://www.ncbi.nlm.nih.gov/bioproject/PRJNA1071080).

## Abbreviations

3'UTR: 3'-Untranslated region
AxeAma: Axenic amastigotes
FPKM: Fragments per kilobase of transcript per million mapped reads
GO: Gene Ontology
HMM: Hidden Markov model
IQR: Interquartile range
MϕAma: Macrophage-derived amastigotes
ORF: Open reading frame
PCA: Principal component analysis
PRED-A-TERM: PREDicting poly(A) sites and TERMinal splice junctions
Pro: Promastigotes
PTR: Posttranscriptional regulation
PTU: Posttranscriptional unit
SD: Standard deviation
SIDER: Short interspersed degenerated retroposon
SII: Signature II sequence of SIDER2
SSR: Strand-switch region
TE: Transposable element
TSR: Transcription start region
