# Supplementary figures and images for: Evolutionary divergent clusters of transcribed extinct truncated retroposons drive low mRNA expression and developmental regulation in the protozoan Leishmania
Source: BMC Biol. 2024 Oct 29;22:249. doi: 10.1186/s12915-024-02051-4 (PMC11520807; doi:10.1186/s12915-024-02051-4)

A

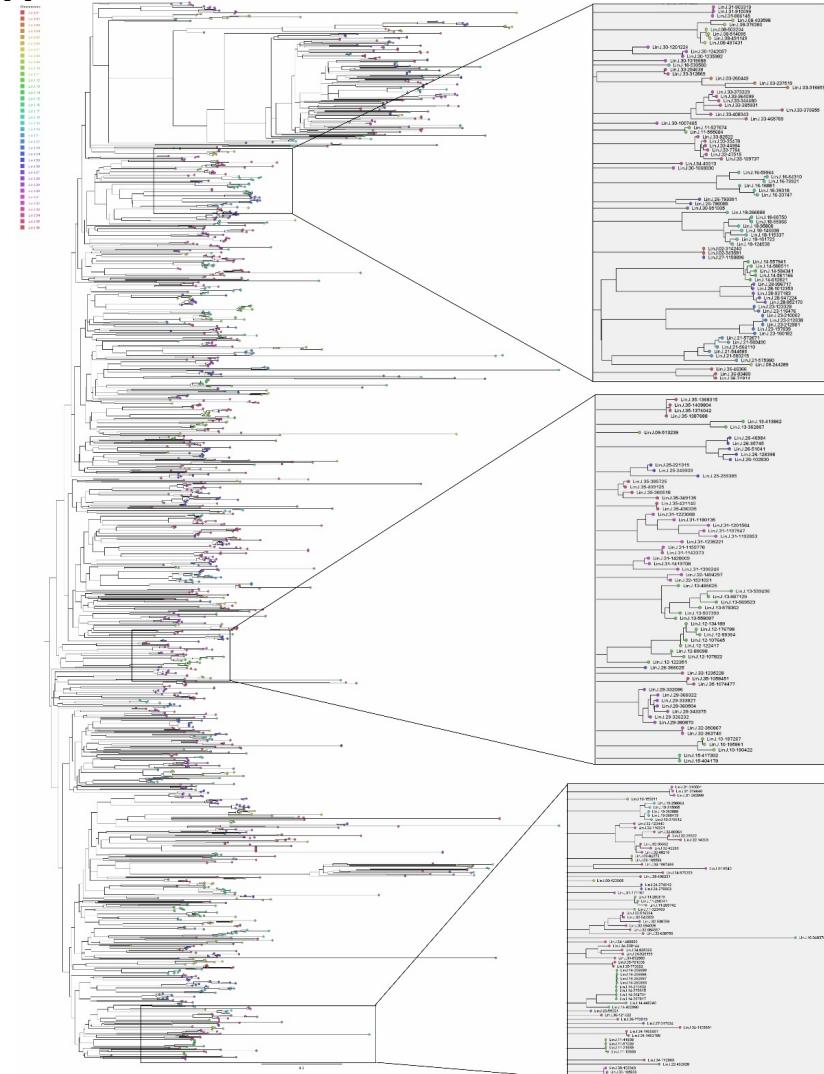

B

Chromosome

- LinJ.14
- LinJ.16
- LinJ.20
- LinJ.22
- LinJ.27

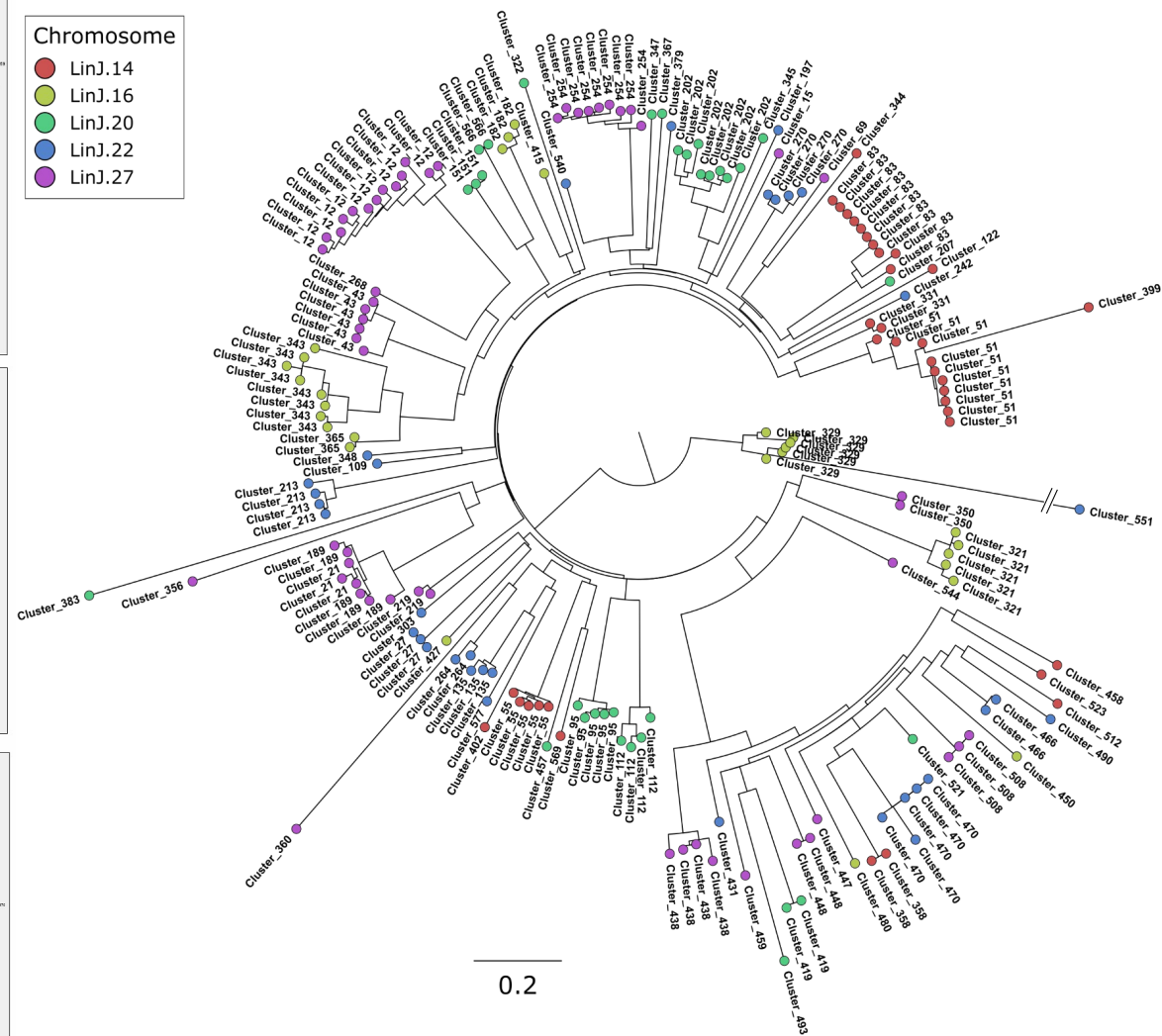

Supplement: Supplementary file 2 — Additional file 2: Figs. S1-S7. Fig. S1. Nucleotide composition of SIDER2 consensus and their hallmark 79-nt signature II sequence. Jalview alignment was performed on the SIDER2 consensus sequence encompassing 536 bp. Within sense SIDER2s, a conserved 79-nt signature (Signature II/SII) resides at the 5'-end, while an A-rich tail typically at the 3'-end (A left). The antisense SIDER2 is represented as the reverse complement of the sense element, featuring a T-rich stretch at the 3'-end and the SII at the 5'-end (A right). B-C Multiple sequence alignments of SIDER2 sequences to the SII consensus using hmmalign and represented by JALVIEW. Signature II consensus or its reverse complement (RC) aligned sequences are shown. The black consensus bars below the alignments show the frequency of the most conserved base indicated underneath. Five SIDER2 sequences within each HMMER group (Groups 1-5) (B) and 25 antisense SIDER2 sequences (C) shown here were randomly selected. Fig. S2. Phylogenetic and CD-Hit-Est analyses of L. infantum SIDER2 sequences. A The unrooted maximum likelihood phylogenetic tree was made using IQ-TREE (v.2.1.2) model GTR + I + G1 of 1448 SIDER2 sequences aligned by MAFFT (v.7.471). SIDER2 sequences were colored by the chromosome where they are located. Three sections were enlarged for better resolution. Tips in the enlarged boxes were named by chromosome followed by the start position of SIDER2. B Concordance between the phylogenetic and CD-Hit-Est analyses. An unrooted maximum likelihood tree generated as indicated in (A) depicted 189 SIDER2 sequences from five chromosomes (LinJ.14, LinJ.16, LinJ.20, LinJ.22 and LinJ.27) aligned by MAFFT (v.7.471). Tips on the tree were color-coded according to their respective chromosomes. Each SIDER2 element is labeled with its corresponding CD-Hit-Est cluster (see Methods). The same tree is represented in Fig. 2B, but with the tips labeled by the LinJ transcripts harboring SIDER2 elements. Fig. S3. Heatmap of regions [file 12915_2024_2051_MOESM2_ESM.zip › Additional file 2 Fig. S2.pdf]

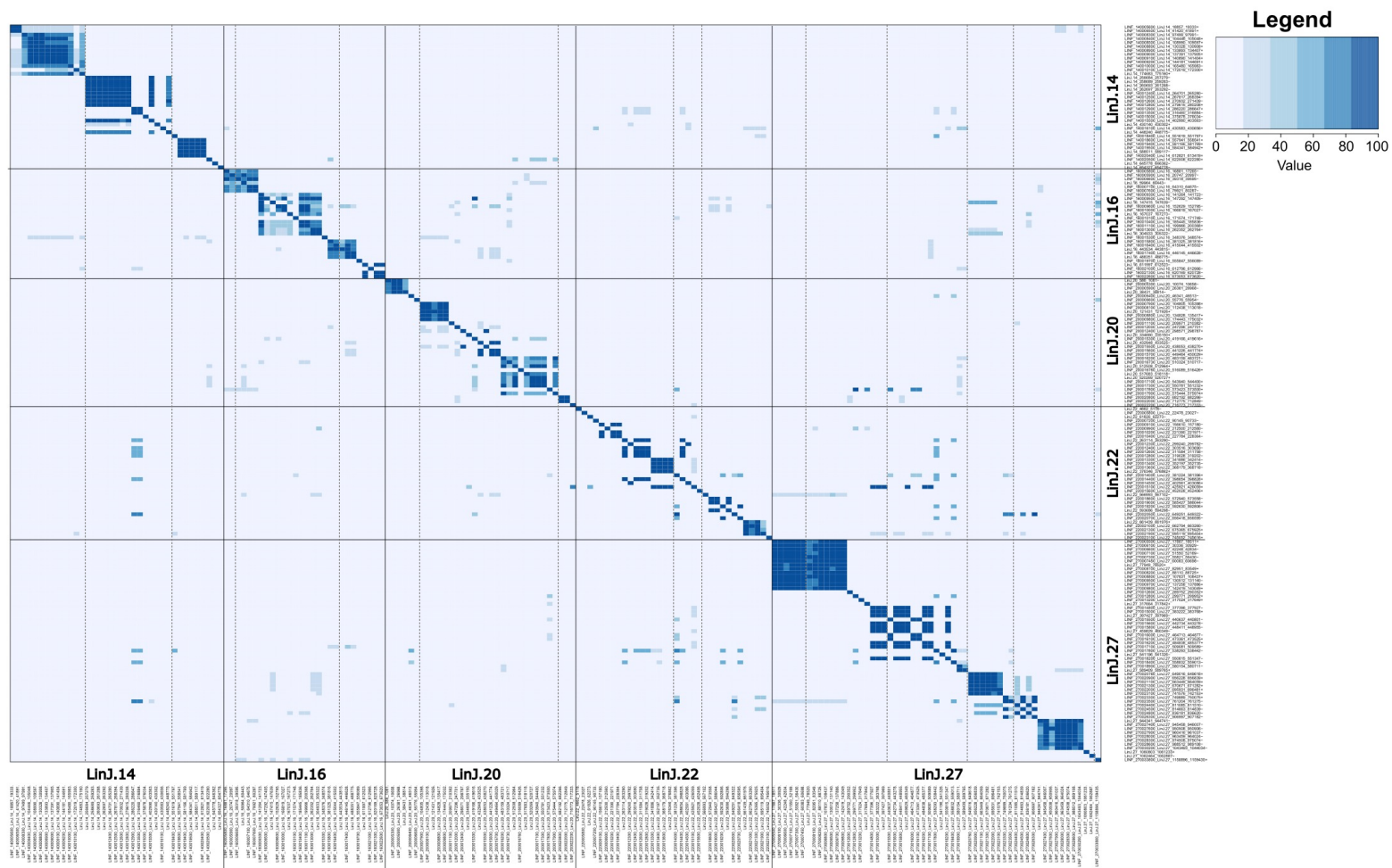

Supplement: Supplementary file 2 — Additional file 2: Figs. S1-S7. Fig. S1. Nucleotide composition of SIDER2 consensus and their hallmark 79-nt signature II sequence. Jalview alignment was performed on the SIDER2 consensus sequence encompassing 536 bp. Within sense SIDER2s, a conserved 79-nt signature (Signature II/SII) resides at the 5'-end, while an A-rich tail typically at the 3'-end (A left). The antisense SIDER2 is represented as the reverse complement of the sense element, featuring a T-rich stretch at the 3'-end and the SII at the 5'-end (A right). B-C Multiple sequence alignments of SIDER2 sequences to the SII consensus using hmmalign and represented by JALVIEW. Signature II consensus or its reverse complement (RC) aligned sequences are shown. The black consensus bars below the alignments show the frequency of the most conserved base indicated underneath. Five SIDER2 sequences within each HMMER group (Groups 1-5) (B) and 25 antisense SIDER2 sequences (C) shown here were randomly selected. Fig. S2. Phylogenetic and CD-Hit-Est analyses of L. infantum SIDER2 sequences. A The unrooted maximum likelihood phylogenetic tree was made using IQ-TREE (v.2.1.2) model GTR + I + G1 of 1448 SIDER2 sequences aligned by MAFFT (v.7.471). SIDER2 sequences were colored by the chromosome where they are located. Three sections were enlarged for better resolution. Tips in the enlarged boxes were named by chromosome followed by the start position of SIDER2. B Concordance between the phylogenetic and CD-Hit-Est analyses. An unrooted maximum likelihood tree generated as indicated in (A) depicted 189 SIDER2 sequences from five chromosomes (LinJ.14, LinJ.16, LinJ.20, LinJ.22 and LinJ.27) aligned by MAFFT (v.7.471). Tips on the tree were color-coded according to their respective chromosomes. Each SIDER2 element is labeled with its corresponding CD-Hit-Est cluster (see Methods). The same tree is represented in Fig. 2B, but with the tips labeled by the LinJ transcripts harboring SIDER2 elements. Fig. S3. Heatmap of regions [file 12915_2024_2051_MOESM2_ESM.zip › Additional file 2 Fig. S3.pdf]

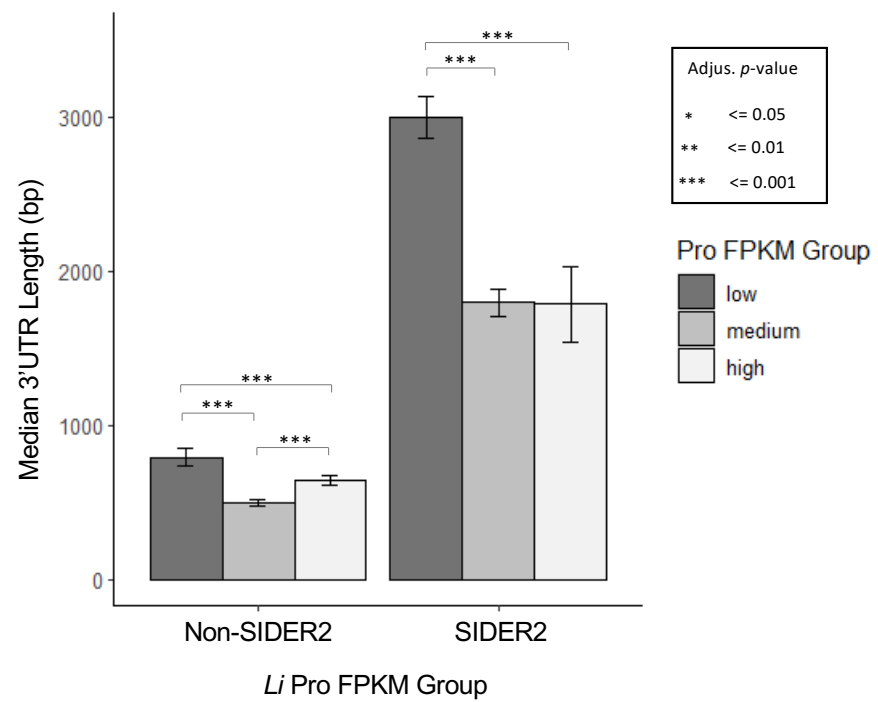

Supplement: Supplementary file 2 — Additional file 2: Figs. S1-S7. Fig. S1. Nucleotide composition of SIDER2 consensus and their hallmark 79-nt signature II sequence. Jalview alignment was performed on the SIDER2 consensus sequence encompassing 536 bp. Within sense SIDER2s, a conserved 79-nt signature (Signature II/SII) resides at the 5'-end, while an A-rich tail typically at the 3'-end (A left). The antisense SIDER2 is represented as the reverse complement of the sense element, featuring a T-rich stretch at the 3'-end and the SII at the 5'-end (A right). B-C Multiple sequence alignments of SIDER2 sequences to the SII consensus using hmmalign and represented by JALVIEW. Signature II consensus or its reverse complement (RC) aligned sequences are shown. The black consensus bars below the alignments show the frequency of the most conserved base indicated underneath. Five SIDER2 sequences within each HMMER group (Groups 1-5) (B) and 25 antisense SIDER2 sequences (C) shown here were randomly selected. Fig. S2. Phylogenetic and CD-Hit-Est analyses of L. infantum SIDER2 sequences. A The unrooted maximum likelihood phylogenetic tree was made using IQ-TREE (v.2.1.2) model GTR + I + G1 of 1448 SIDER2 sequences aligned by MAFFT (v.7.471). SIDER2 sequences were colored by the chromosome where they are located. Three sections were enlarged for better resolution. Tips in the enlarged boxes were named by chromosome followed by the start position of SIDER2. B Concordance between the phylogenetic and CD-Hit-Est analyses. An unrooted maximum likelihood tree generated as indicated in (A) depicted 189 SIDER2 sequences from five chromosomes (LinJ.14, LinJ.16, LinJ.20, LinJ.22 and LinJ.27) aligned by MAFFT (v.7.471). Tips on the tree were color-coded according to their respective chromosomes. Each SIDER2 element is labeled with its corresponding CD-Hit-Est cluster (see Methods). The same tree is represented in Fig. 2B, but with the tips labeled by the LinJ transcripts harboring SIDER2 elements. Fig. S3. Heatmap of regions [file 12915_2024_2051_MOESM2_ESM.zip › Additional file 2 Fig. S4.pdf]

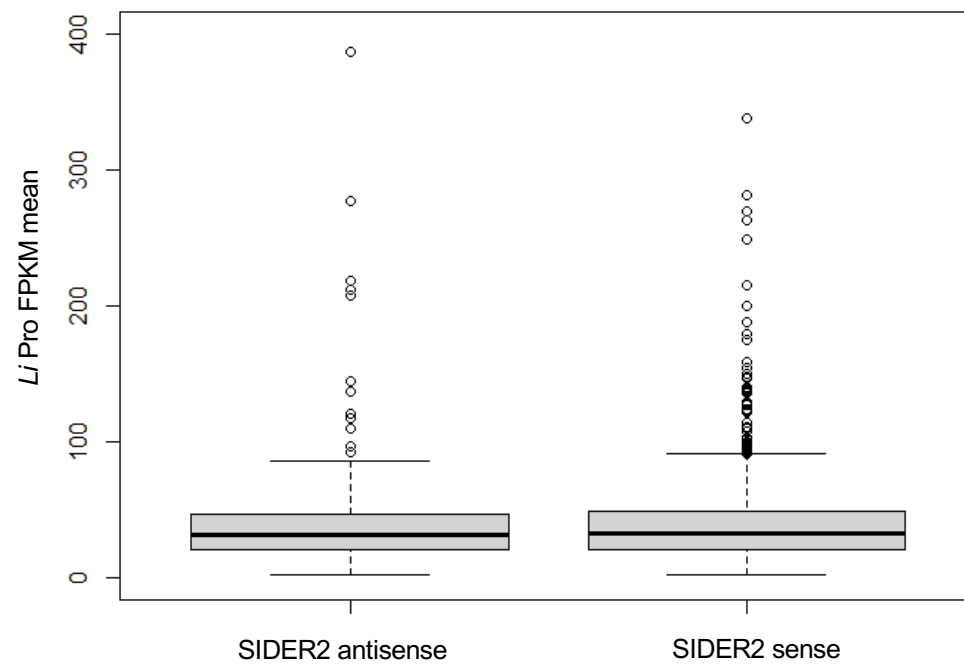

Supplement: Supplementary file 2 — Additional file 2: Figs. S1-S7. Fig. S1. Nucleotide composition of SIDER2 consensus and their hallmark 79-nt signature II sequence. Jalview alignment was performed on the SIDER2 consensus sequence encompassing 536 bp. Within sense SIDER2s, a conserved 79-nt signature (Signature II/SII) resides at the 5'-end, while an A-rich tail typically at the 3'-end (A left). The antisense SIDER2 is represented as the reverse complement of the sense element, featuring a T-rich stretch at the 3'-end and the SII at the 5'-end (A right). B-C Multiple sequence alignments of SIDER2 sequences to the SII consensus using hmmalign and represented by JALVIEW. Signature II consensus or its reverse complement (RC) aligned sequences are shown. The black consensus bars below the alignments show the frequency of the most conserved base indicated underneath. Five SIDER2 sequences within each HMMER group (Groups 1-5) (B) and 25 antisense SIDER2 sequences (C) shown here were randomly selected. Fig. S2. Phylogenetic and CD-Hit-Est analyses of L. infantum SIDER2 sequences. A The unrooted maximum likelihood phylogenetic tree was made using IQ-TREE (v.2.1.2) model GTR + I + G1 of 1448 SIDER2 sequences aligned by MAFFT (v.7.471). SIDER2 sequences were colored by the chromosome where they are located. Three sections were enlarged for better resolution. Tips in the enlarged boxes were named by chromosome followed by the start position of SIDER2. B Concordance between the phylogenetic and CD-Hit-Est analyses. An unrooted maximum likelihood tree generated as indicated in (A) depicted 189 SIDER2 sequences from five chromosomes (LinJ.14, LinJ.16, LinJ.20, LinJ.22 and LinJ.27) aligned by MAFFT (v.7.471). Tips on the tree were color-coded according to their respective chromosomes. Each SIDER2 element is labeled with its corresponding CD-Hit-Est cluster (see Methods). The same tree is represented in Fig. 2B, but with the tips labeled by the LinJ transcripts harboring SIDER2 elements. Fig. S3. Heatmap of regions [file 12915_2024_2051_MOESM2_ESM.zip › Additional file 2 Fig. S5.pdf]

**A**

Chr 1 2 3 4 5 6 7 8 9 10 11 12 13 14 15 16 17 18 19 20 21 22 23 24 25 26 27 28 29 30 31 32 33 34 35 36

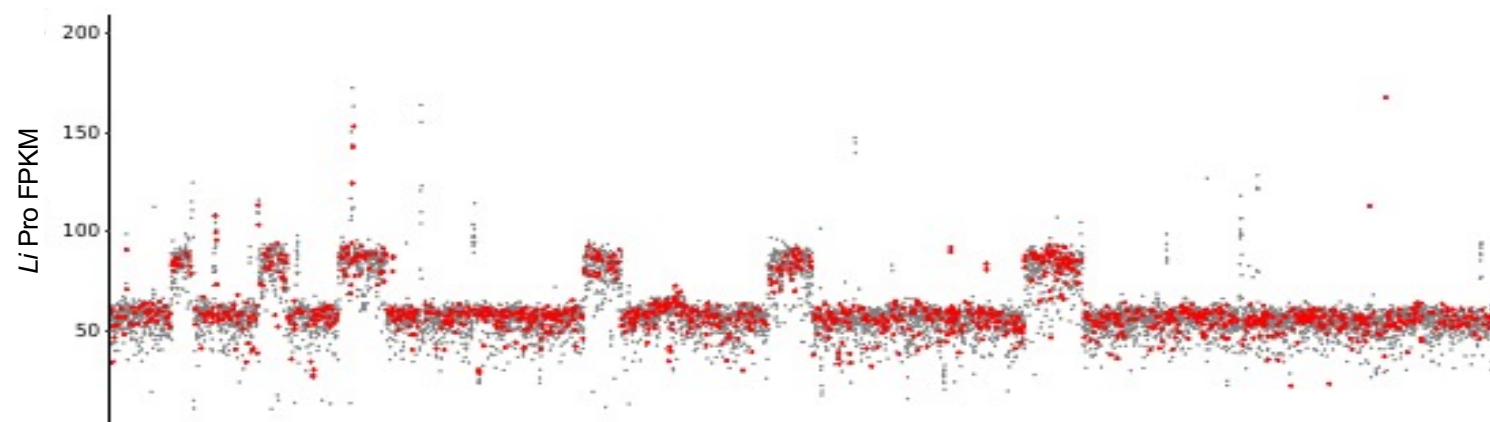**B**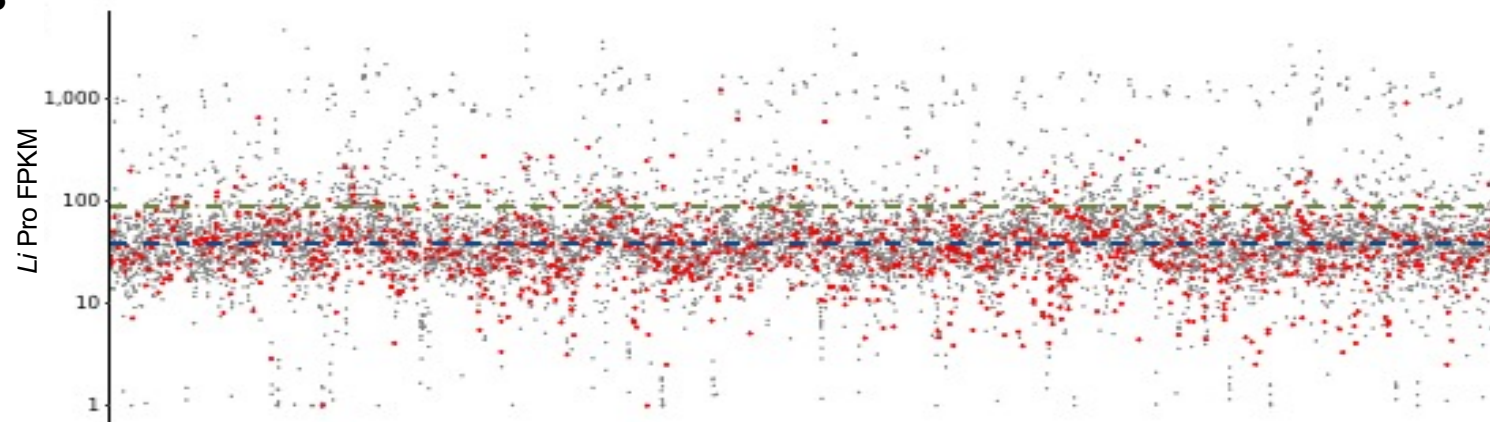

Supplement: Supplementary file 2 — Additional file 2: Figs. S1-S7. Fig. S1. Nucleotide composition of SIDER2 consensus and their hallmark 79-nt signature II sequence. Jalview alignment was performed on the SIDER2 consensus sequence encompassing 536 bp. Within sense SIDER2s, a conserved 79-nt signature (Signature II/SII) resides at the 5'-end, while an A-rich tail typically at the 3'-end (A left). The antisense SIDER2 is represented as the reverse complement of the sense element, featuring a T-rich stretch at the 3'-end and the SII at the 5'-end (A right). B-C Multiple sequence alignments of SIDER2 sequences to the SII consensus using hmmalign and represented by JALVIEW. Signature II consensus or its reverse complement (RC) aligned sequences are shown. The black consensus bars below the alignments show the frequency of the most conserved base indicated underneath. Five SIDER2 sequences within each HMMER group (Groups 1-5) (B) and 25 antisense SIDER2 sequences (C) shown here were randomly selected. Fig. S2. Phylogenetic and CD-Hit-Est analyses of L. infantum SIDER2 sequences. A The unrooted maximum likelihood phylogenetic tree was made using IQ-TREE (v.2.1.2) model GTR + I + G1 of 1448 SIDER2 sequences aligned by MAFFT (v.7.471). SIDER2 sequences were colored by the chromosome where they are located. Three sections were enlarged for better resolution. Tips in the enlarged boxes were named by chromosome followed by the start position of SIDER2. B Concordance between the phylogenetic and CD-Hit-Est analyses. An unrooted maximum likelihood tree generated as indicated in (A) depicted 189 SIDER2 sequences from five chromosomes (LinJ.14, LinJ.16, LinJ.20, LinJ.22 and LinJ.27) aligned by MAFFT (v.7.471). Tips on the tree were color-coded according to their respective chromosomes. Each SIDER2 element is labeled with its corresponding CD-Hit-Est cluster (see Methods). The same tree is represented in Fig. 2B, but with the tips labeled by the LinJ transcripts harboring SIDER2 elements. Fig. S3. Heatmap of regions [file 12915_2024_2051_MOESM2_ESM.zip › Additional file 2 Fig. S6.pdf]

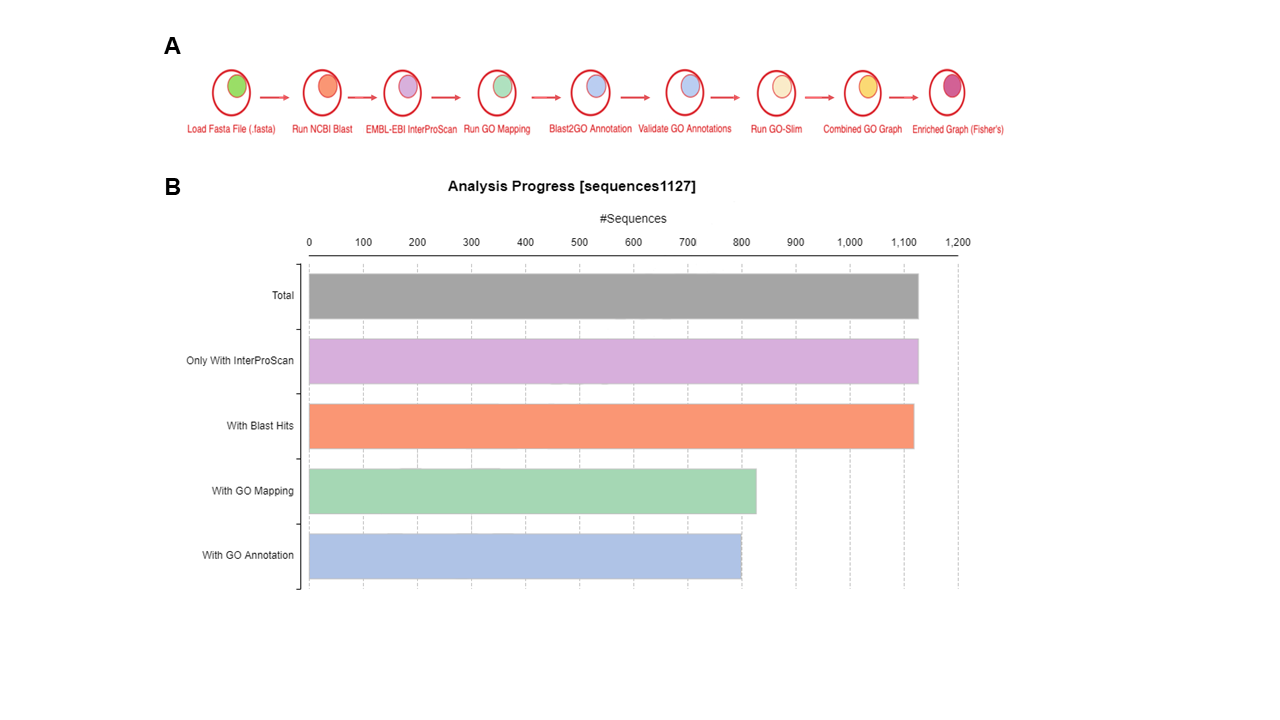

Supplement: Supplementary file 2 — Additional file 2: Figs. S1-S7. Fig. S1. Nucleotide composition of SIDER2 consensus and their hallmark 79-nt signature II sequence. Jalview alignment was performed on the SIDER2 consensus sequence encompassing 536 bp. Within sense SIDER2s, a conserved 79-nt signature (Signature II/SII) resides at the 5'-end, while an A-rich tail typically at the 3'-end (A left). The antisense SIDER2 is represented as the reverse complement of the sense element, featuring a T-rich stretch at the 3'-end and the SII at the 5'-end (A right). B-C Multiple sequence alignments of SIDER2 sequences to the SII consensus using hmmalign and represented by JALVIEW. Signature II consensus or its reverse complement (RC) aligned sequences are shown. The black consensus bars below the alignments show the frequency of the most conserved base indicated underneath. Five SIDER2 sequences within each HMMER group (Groups 1-5) (B) and 25 antisense SIDER2 sequences (C) shown here were randomly selected. Fig. S2. Phylogenetic and CD-Hit-Est analyses of L. infantum SIDER2 sequences. A The unrooted maximum likelihood phylogenetic tree was made using IQ-TREE (v.2.1.2) model GTR + I + G1 of 1448 SIDER2 sequences aligned by MAFFT (v.7.471). SIDER2 sequences were colored by the chromosome where they are located. Three sections were enlarged for better resolution. Tips in the enlarged boxes were named by chromosome followed by the start position of SIDER2. B Concordance between the phylogenetic and CD-Hit-Est analyses. An unrooted maximum likelihood tree generated as indicated in (A) depicted 189 SIDER2 sequences from five chromosomes (LinJ.14, LinJ.16, LinJ.20, LinJ.22 and LinJ.27) aligned by MAFFT (v.7.471). Tips on the tree were color-coded according to their respective chromosomes. Each SIDER2 element is labeled with its corresponding CD-Hit-Est cluster (see Methods). The same tree is represented in Fig. 2B, but with the tips labeled by the LinJ transcripts harboring SIDER2 elements. Fig. S3. Heatmap of regions [file 12915_2024_2051_MOESM2_ESM.zip › Additional file 2 Fig. S7.tif]
